# Measuring the effects of COVID-19-related disruption on dengue transmission in southeast Asia and Latin America: a statistical modelling study

**DOI:** 10.1016/S1473-3099(22)00025-1

**Published:** 2022-05

**Authors:** Yuyang Chen, Naizhe Li, José Lourenço, Lin Wang, Bernard Cazelles, Lu Dong, Bingying Li, Yang Liu, Mark Jit, Nikos I Bosse, Sam Abbott, Raman Velayudhan, Annelies Wilder-Smith, Huaiyu Tian, Oliver J Brady, CMMID COVID-19 Working Group, Simon R Procter, Kerry LM Wong, Joel Hellewell, Nicholas G Davies, Christopher I Jarvis, Ciara V McCarthy, Graham Medley, Sophie R Meakin, Alicia Rosello, Emilie Finch, Rachel Lowe, Carl A B Pearson, Samuel Clifford, Billy J Quilty, Stefan Flasche, Hamish P Gibbs, Lloyd A C Chapman, Katherine E. Atkins, David Hodgson, Rosanna C Barnard, Timothy W Russell, Petra Klepac, Yalda Jafari, Rosalind M Eggo, Paul Mee, Matthew Quaife, Akira Endo, Sebastian Funk, Stéphane Hué, Adam J Kucharski, W John Edmunds, Kathleen O'Reilly, Rachael Pung, C Julian Villabona-Arenas, Amy Gimma, Kaja Abbas, Kiesha Prem, Gwenan M Knight, Fiona Yueqian Sun, William Waites, James D Munday, Mihaly Koltai, Frank G Sandmann, Damien C Tully

**Affiliations:** a State Key Laboratory of Remote Sensing Science, Center for Global Change and Public Health, College of Global Change and Earth System Science, Beijing Normal University, Beijing, China; b MOE Key Laboratory for Biodiversity Science and Ecological Engineering, College of Life Sciences, Beijing Normal University, Beijing, China; c Biosystems and Integrative Sciences Institute, University of Lisbon, Lisbon, Portugal; d Department of Genetics, University of Cambridge, Cambridge, UK; e Mathematical Modelling of Infectious Diseases Unit, Institut Pasteur, UMR2000, CNRS, Paris, France; f Institut de Biologie de l'École Normale Supérieure UMR8197, Eco-Evolutionary Mathematics, École Normale Supérieure, Paris, France; g Unité Mixte Internationnale 209, Mathematical and Computational Modeling of Complex Systems, Sorbonne Université, Paris, France; h Centre for Mathematical Modelling of Infectious Diseases, London School of Hygiene & Tropical Medicine, London, UK; i Department of Infectious Disease Epidemiology, Faculty of Epidemiology and Population Health, London School of Hygiene & Tropical Medicine, London, UK; j Department of Disease Control, London School of Hygiene & Tropical Medicine, London, UK; k Department of Control of Neglected Tropical Diseases, WHO, Geneva, Switzerland; l Heidelberg Institute of Global Health, University of Heidelberg, Heidelberg, Germany

## Abstract

**Background:**

The COVID-19 pandemic has resulted in unprecedented disruption to society, which indirectly affects infectious disease dynamics. We aimed to assess the effects of COVID-19-related disruption on dengue, a major expanding acute public health threat, in southeast Asia and Latin America.

**Methods:**

We assembled data on monthly dengue incidence from WHO weekly reports, climatic data from ERA5, and population variables from WorldPop for 23 countries between January, 2014 and December, 2019 and fit a Bayesian regression model to explain and predict seasonal and multi-year dengue cycles. We compared model predictions with reported dengue data January to December, 2020, and assessed if deviations from projected incidence since March, 2020 are associated with specific public health and social measures (from the Oxford Coronavirus Government Response Tracer database) or human movement behaviours (as measured by Google mobility reports).

**Findings:**

We found a consistent, prolonged decline in dengue incidence across many dengue-endemic regions that began in March, 2020 (2·28 million cases in 2020 *vs* 4·08 million cases in 2019; a 44·1% decrease). We found a strong association between COVID-19-related disruption (as measured independently by public health and social measures and human movement behaviours) and reduced dengue risk, even after taking into account other drivers of dengue cycles including climatic and host immunity (relative risk 0·01–0·17, p<0·01). Measures related to the closure of schools and reduced time spent in non-residential areas had the strongest evidence of association with reduced dengue risk, but high collinearity between covariates made specific attribution challenging. Overall, we estimate that 0·72 million (95% CI 0·12–1·47) fewer dengue cases occurred in 2020 potentially attributable to COVID-19-related disruption.

**Interpretation:**

In most countries, COVID-19-related disruption led to historically low dengue incidence in 2020. Continuous monitoring of dengue incidence as COVID-19-related restrictions are relaxed will be important and could give new insights into transmission processes and intervention options.

**Funding:**

National Key Research and Development Program of China and the Medical Research Council.

## Introduction

Dengue is a major cause of acute morbidity in over 120 countries worldwide and is one of the few infectious diseases to show sustained increases year on year.^1^ Countries in the Americas and southeast Asia regions are worst affected and routinely account for the majority of global cases.^2^ In 2020, more than 2 million cases were reported from these regions, which is substantially lower than the 5·2 million recorded in 2019, or many previous years.^3^, ^4^

The COVID-19 pandemic has led to substantial societal disruption in 2020, including changes to human movement behaviours and the closure of specific venues and modes of transport where humans often mixed, through government-imposed public health and social measures.^5^ Dengue virus is transmitted to humans by *Aedes* species mosquitoes and transmission is closely linked to changes in weather, the natural and built environment, and human mobility.^6^ Declines in human mobility—either voluntarily or through restrictions (ie, public health and social measures)—could reduce dengue virus transmission, but might also disrupt vector control and thus increase dengue virus transmission. The effects of COVID-19-related disruption might also depend on the relative importance of inside versus outside the home for dengue virus transmission. ^7^ Although overall cases of dengue declined in 2020, worse than average dengue incidence was reported in Peru^8^ and Singapore for 2020.^9^ Given that 2019 saw the largest global dengue outbreak in history, observing and attributing the effects of COVID-19 disruption is complicated.^2^ Some countries might be experiencing the continuance of this global dengue epidemic, while others might be experiencing below average transmission due to the build-up of immunity. Finally, concerns have been raised about under-reporting of dengue case statistics in 2020 given reduced treatment-seeking rates, higher potential for clinical misdiagnosis, and reduced availability of laboratory testing for dengue.^10^

**Research in context**

**Evidence before this study**

Previous studies have shown that human movement, heterogeneity in environmental risk, and mosquito control practices all strongly influence the transmission of dengue virus. Restrictions put in place in response to the COVID-19 pandemic led to substantial changes in how people move, where they spend time, and the continuity of disease control programmes, but the net effect on dengue remains unclear. We searched PubMed for studies published between database inception and April 4, 2021, without language restrictions, using the search terms “(COVID-19 OR coronavirus OR SARS-CoV-2) AND (lockdown OR interventions OR restriction OR human mobility) AND (dengue* OR DENV*)”. We also searched WHO Weekly Report and government websites for dengue case data reported for countries in Latin America and southeast Asia. Although 15 studies warned about the risk of COVID-19 exacerbating dengue transmission and the subsequent pressure on intensive care resources, only three studies analysed dengue and COVID-19 data from 2020. Among the three studies that have looked for associations between COVID-19 restrictions and dengue, findings have been mixed—with protective effects, enhancing effects, and no significant effects seen in different countries. Assessing the effect of the COVID-19 pandemic on dengue is challenging due to the high immunity levels against dengue caused by an unusually large global dengue outbreak in 2019 and previously incomplete dengue datasets from 2020.

**Added value of this study**

To our knowledge, this study is the first to analyse dengue data throughout 2020 from 23 countries spanning the main dengue endemic regions of Latin America and southeast Asia. Our findings show that there is a consistent association between various measures of COVID-19-related disruption and reduced dengue transmission that cannot be explained by seasonal or extra-seasonal dengue cycles or underreporting. Although attributing change to specific restrictions or behaviours was restricted by collinearity, we present evidence that suggests specific roles for schools and other commonly visited non-residential venues.

**Implications of all the available evidence**

This combined evidence base emphasises the importance of high-traffic, high-mixing venues for dengue transmission and could lead to new interventions and targeting strategies. Although we are unlikely to ever see 2020-like restrictions used to control dengue outbreaks, targeted testing and mosquito control based on patient-reported recent movements could offer new approaches for a disease that continues to evade control by existing approaches.

For dengue, the COVID-19 pandemic provides a unique opportunity to understand how different environments and human movement contribute to transmission and could lead to new interventions and strategies after the public health and social measures are relaxed.^11^

We aimed to conduct the first multi-continent assessment of the effects of public health and social measures on dengue incidence using data from 23 countries, with the goal of quantifying the strength and magnitude of associations between COVID-19-related disruption and dengue virus transmission dynamics.

## Methods

### Study design and participants

**Figure 1:**
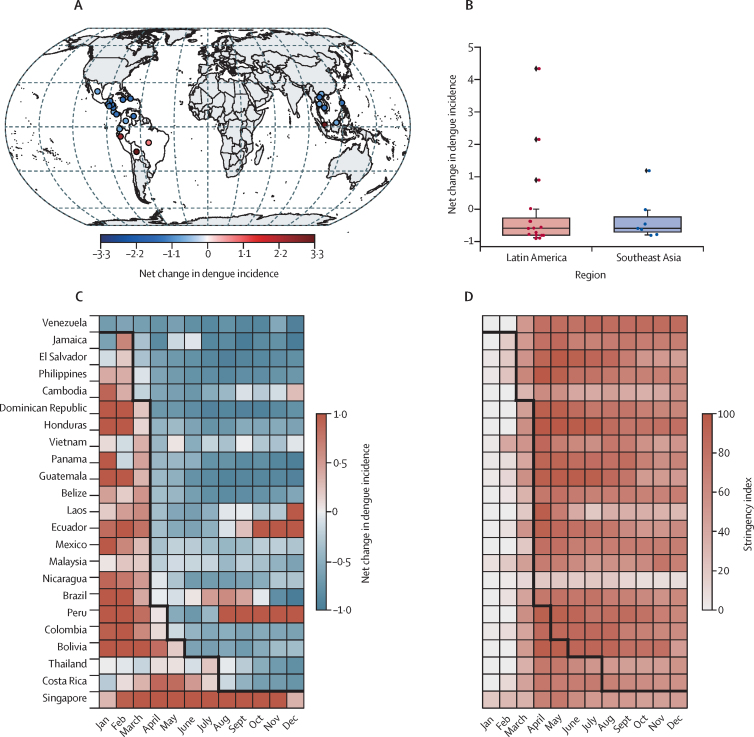
Dengue incidence and government interventions in Latin America and southeast Asia in 2020 versus 2014–19 (A) Relative change ratio of annual dengue incidence in 2020 versus the mean incidence in 2014–19. (B) Distribution of relative change ratio of annual dengue incidence for each country in 2020 versus 2019. The boxplot displays 2·5th, 25th, 50th,75th and 97·5th percentiles. (C) The relative change ratio of monthly dengue incidence in 2020 relative to the monthly mean incidence in 2014–19. (D) Change of government stringency index against COVID-19 in 2020. The black line represents the beginning of a consistent dengue incidence decline in 2020 versus the monthly mean in 2014–19.

The study covers 23 countries (16 in Latin America and 7 in southeast Asia; listed in figure 1) located between 30° N and 30° S that reported at least 2000 cases per year during 2014–20. We collected the monthly number of dengue cases in 2014–20 as reported by WHO weekly reports.^3^, ^4^ Cases comprise a mixture of suspected and diagnostic test-confirmed cases with case definition and surveillance quality differing between countries, but with increased consistency when comparing within a country over time.^12^

### Climate and population data

We obtained air temperature at 2 m above the earth's surface, surface temperature, relative humidity, convective precipitation, and total precipitation during 2014–20 from ERA5 monthly averaged reanalysis data with a resolution of 0·1° × 0·1°.^13^ The climate variables chosen in our analysis have proven associations with dengue transmission.^14^ Environmental covariates were averaged across countries as populaton-weighted averages. We used population-weighted means based on WorldPop population data (appendix p 5).^15^, ^16^

### Restrictions and human mobility data

We independently tested for associations between dengue risk and two different measures of COVID-19-related disruption: announced containment and closure policies (hereafter public health and social measures) and observed changes in human movement behaviours. We extracted data on public health and social measures from the Oxford Coronavirus Government Response Tracer (OxCGRT) project.^17^ This included eight containment and closure public health and social measures (school closing, workplace closing, cancelling of public events, restrictions on gathering sizes, closing public transport, stay at home requirements, restrictions on internal movement, and international travel controls) in addition to an overall stringency index based on public health and social measures and other restriction indicators.

Because individual public health and social measures can vary in intensity and domestic geographical scope, we used OxCGRT proposed methods^17^ to convert the original ordinal data for each public health and social measures to eight continuous sub-index scores. These public health and social measures indices take values between 0 and 100 and allow international comparability of public health and social measures, taking into account both their extent and intensity. A higher score indicates a more stringent, more geographically comprehensive COVID-19 response policy (0 for no response policy and 100 for the most stringent response policy). Socio-geographical human mobility data across countries were obtained from Google Mobility Reports, which included human movement behaviour metrics on time spent in six different location types (residential, workplace, transit stations, parks, grocery and pharmacy, and retail and recreation). The baseline is the median value for the corresponding day of the week during the first 5 weeks of 2020 (Jan 3–Feb 6). For the purpose of our analysis, we assume all human movement behaviour variables take the value of 100% before Feb 7, 2020.

### Collinearity analysis

Because there could be a high degree of collinearity between different public health and social measures or human movement behaviours, we used Pearson correlation analysis and hierarchical clustering analysis, using Ward's method,^18^ to compare the similarity of the timeseries of eight public health and social measures and six human movement behaviour variables (no lagged terms included) across all countries. We used Euclidean distance to distinguish clusters of multiple variables and assess variable similarity following a previous study.^19^ We used multi-scale bootstrapping (n=10 000) to test the statistical significance of the identified clusters, defined using approximate unbiased p values less than 0·05 in the “pvclust” R package (version 2.2).

### Under-reporting analysis

To assess under-reporting, we calculated country-specific annual case fatality rates (deaths / cases). We hypothesise that if COVID-19 has substantially impacted dengue reporting, this disruption will disproportionately affect less severe cases, with severe and fatal cases still warranting emergency care, diagnosis, and reporting even during peak disruption. This scenario would result in excessively high case fatality rates in 2020 versus years.

### Statistical analysis

First, a historical model was fitted to monthly case counts before 2020 (January, 2014 to December, 2019) in 23 countries:

$$\begin{array}{ll} \gamma_{c,t}|\text{dengue}{\text{case}}_{c,t} & \kappa \sim NegBin(denguecase_{c,t},\kappa )\\ \log(denguecase_{c,t}) & =\text{NS}(\text{climate}{\text{factors}}_{c,t},\mathrm{var}df,lagdf)\\ & +\beta_{c,m(t)}+\phi_{c,a,(t)}+\mu_{c,a,(t)}\\ & +\text{annual}\text{anomaly}[c,a(t)-1]\\ & +offset[{\text{population}}_{\text{c},\text{a}(\text{t})}] \end{array}$$

where *y*_c,t_ is the monthly number of cases in the respective country. We used a negative binomial distribution for the response variable (dengue cases) to account for overdispersion (κ) of case counts. We included a distributed lag non-linear model formulation using natural cubic spline (NS) in R packages dlnm and splines to quantify the non-linear relationship between each climate factor (*var df*) with changing dengue risk over different lag periods 0–3 months (*lag df*; appendix pp 4–7).^20^, ^21^ The difference between cases reported in the previous year and mean long-term annual average (over years 2014–19) (annual anomaly_[c,a(t) – 1]_) was introduced to account for inter-annual immunity changes, where *a(t)* = 2014,…,2019. We included structured random effects to account for spatial, seasonal, and extra-seasonal variations in unknown and unmeasurable factors (such as differences in health care, vector control, and human mobility). We fit a cyclic first-order monthly random walk with no discontinuity between December in year_a(t) – 1_ and January in year_a(t)_, *ß*_c, m(t)_, for each country to account for seasonality, where *m*_(t)_ = 1,…12 (January to December). We also included a modified Besag-York-Mollié model,^22^ which consists of one precision parameter and one mixing parameter that determines the relative contribution of spatially structured (*μ*_c, a(t)_) and unstructured (φ_c, a(t)_) random effects. We use a penalised complexity prior approach for the precision *t* = 1 / σ^2^, so that Pr(1 / √*t* >0·5) = 0·01.^23^ Population size was included as an offset variable.

Model parameters were estimated using integrated nested laplace approximation in a Bayesian framework with flat uninformative priors. All combinations of climatic, economic, and immunity covariates were tested in different mode formulations, all of which included spatiotemporal random effects. We used deviance information criterion and mean cross-validated log score (on a repeated leave 1 month per country out hold-out set) to test model explanatory and predictive power.^24^ To test long-term (12 month) predictive performance, we also tested the model's ability to predict monthly dengue incidence in 2018 when fit to data for 2015–17.

A second non-Bayesian intervention model was fit to monthly dengue data in 2020 (*t*), taking into account the mean predicted case counts from the historical model (on the log scale, ў).

$$\begin{array}{l} \gamma_{c,t}|denguecase_{c,t}\kappa \sim NegBin(denguecase_{c,t},\kappa )\\ \log(denguecase_{c,t})=NS(PHSM_{c,t}orHMB_{c,t},)\\ \mathrm{var}df,lagdf)+offset({\ddot{\gamma }}_{c,t}) \end{array}$$

where PHSM denotes public health and social measures and HMB denotes human mobility behaviour.

Two separate, distributed lag, non-linear models were formulated to test for associations between dengue risk and either public health and social measures or human movement behaviours. Relative risk (RR) estimates for public health and social measures or human movement behaviours were calculated relative to values of 0 or 100%, respectively.

Both univariable and multivariable models were fit to compare the direction and strength of associations at different lags. Multivariable models used both forward and backward selection procedures based on Akaike information criterion as implemented in the mvabund R package.

To estimate the number of dengue cases averted due to general COVID-19-related disruption in 2020, we calculated the difference between observed cases in 2020 and the number of cases predicted by the historical model for that year. To attribute these averted cases to specific public health and social measures and human movement behaviours, we used the RR estimated by the final multivariable intervention model estimated to quantify the monthly prevention fraction using the forward attribution method.^25^ The total prevention fraction was the sum of prevention fraction for 12 months in 2020.

All analyses were performed in R (version 4.1.12).

### Role of the funding source

The funders of the study had no role in study design, data collection, data analysis, data interpretation, or writing of the report.

## Results

19 of 23 countries reported lower dengue incidence in 2020 (cumulative Jan–Dec, 2020) than average (*vs* 2014–19, figure 1A), with exceptions seen in Brazil, Peru, Bolivia, and Singapore. Compared with 2019, incidence decreased by 44·1% across the study area of Latin America and southeast Asia (2·28 million cases in 2020 *vs* 4·08 million cases in 2019), with a 40·2% decrease in Latin America (569·26 to 340·33 cases per 100 000 population) and 58·4% decrease in southeast Asia (297·31 to 123·58 cases per 100 000 population, figure 1B). This decline becomes even more pronounced when comparing incidence from April 2020 onwards (figure 1C); exceptions include Singapore, which saw above average caseloads throughout 2020, and Ecuador, Brazil, and Peru, which had extra-seasonal increases later in the year. At the time of analysis, we were unable to obtain complete (Jan–Dec, 2020) reported dengue case values for several large dengue-endemic countries, including India, Sri Lanka, Nepal, Myanmar, Paraguay, and Indonesia.

These declines occurred at the beginning of the dengue season in many countries, with cases in southeast Asia, Central America, and the Caribbean typically increasing between June and September. Nine of 11 countries in Central America and the Caribbean, and the Philippines in southeast Asia, saw complete suppression of their 2020 dengue season, with most other countries experiencing a much suppressed dengue season (appendix pp 13–14). In countries where public health and social measures began at the peak of the dengue season, such as in South America, sharper than expected declines were seen despite above average incidence earlier in the year (appendix pp 13–14).

These abnormal declines coincide with the introduction of public health and social measures (late March to early April) and the subsequent shift of human movement behaviours towards time spent in residential premises in late March to April (figure 1D; appendix pp 13–18). The observed climate in 2020 was similar to the average of the previous 6 years, with the exception of mildly higher temperatures in Jamaica and lower temperatures in Venezuela (appendix p 19). We found no evidence that this decline in incidence is due to underreporting. If cases were underreported, we would expect to see higher case fatality rates than reported due to reporting of severe cases being less adversely affected than mild cases. Case fatality rates for 2020 were within the range of the previous 6 years for all countries except Venezuela, which had a mild increase in incidence over its 2017 peak (appendix p 15).

To investigate the association between public health and social measures, human movement behaviours, and dengue incidence, we also considered climatic and immunological factors that can also influence seasonal and extra-seasonal dengue cycles. In the historical model fit to data before the COVID-19 epidemic (2014–19), we retained the climate variables of convective precipitation, surface temperature, and short-term and long-term autocorrelation effects (appendix p 8). This model specification resulted in the largest improvements in both within-sample explanatory and out-of-sample predictive performance, with a decrease in deviance information criterion of 76·30 and cross-validated mean log score of 0·033 over a baseline model of spatiotemporal effects (appendix pp 8, 21–24). The model accurately replicated seasonal dynamics in all countries, explained large outbreak years (eg, 2019) globally and prolonged periods of low transmission (eg, 2017–18 in Central America; appendix pp 29, 31), and estimated approximate seasonal dynamics and comparative outbreak size between countries when making predictions up to a year ahead (appendix p 32).

**Figure 2:**
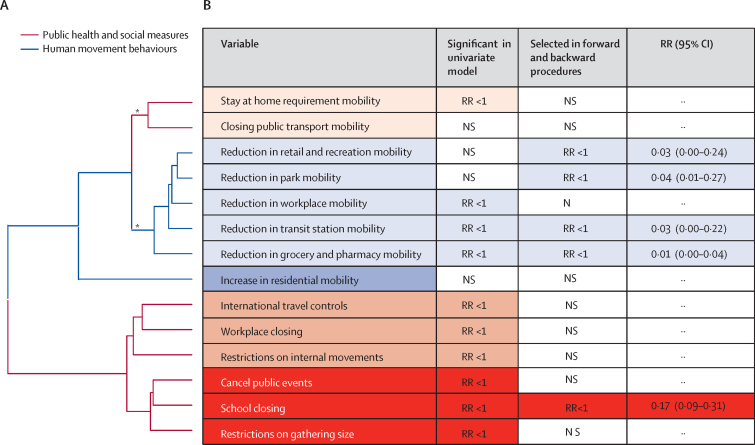
Strength of association between dengue risk and public health and social measures and human mobility behaviours (A) Dendrogram showing the hierarchical clustering of public health and social measures and human mobility behaviours timeseries. The height of nodes connecting two variables on the dendrogram represents the degree of similarity. For example, the school closing variable is more similar to the cancel public events variable than it is to the restrictions on gathering size variable. (B) Data show the strength of evidence of association between dengue risk and either public health and social measures or human movement behaviours. Variables are coloured according to their respective clusters. All columns except the first refer to the multivariable model. For terms with p<0.05, the direction of RR is given. RRs were calculated cumulatively over all lag periods and compare the variables at their strictest (1 and 100%) with baseline pre-pandemic levels, with 95% CIs. *Clusters with approximately unbiased p values larger than 95% are classified as significant clusters. NS=not significant (p>0·05). RR=relative risk.

We used an intervention sub-model to explain the difference between observed case counts in 2020 and predicted case counts in 2020 from the historical model (appendix pp 29, 33). In our univariable intervention model, seven (88%) of eight public health and social measures (except closing public transport) and the composite stringency index showed significant negative correlations with dengue risk. Similarly, three (50%) of six human movement behaviours (except residential, retail or recreation, and park) showed significant positive associations with dengue relative risk (appendix pp 25–26). Although these findings suggest a potential association between one or multiple public health and social measures or human movement behaviours and reduced dengue risk, collinearity prevents us from identifying associations with specific variables when using univariable analyses alone. Therefore, we used a hierarchical cluster analysis to quantify the structural collinearity between public health and social measures and human movement behaviours timeseries. This analysis clustered variables into five distinct clusters, two of which were statistically significant (approximate p>0·05), including the stay at home requirement and closing public transport cluster, and the cluster of all non-residential human movement metrics (figure 2; appendix p 27). For public health and social measures, three variables (cancel public events, school closing, and restrictions on gathering size) were highly colinear (correlation coefficient >0·8; appendix p 28) indicating they were consistently applied at the same time. As expected, the composite stringency index showed high collinearity with all eight of the specific public health and social measures indicators (correlation coefficient range 0·73–0·91; appendix p 28); therefore, we excluded stringency index from subsequent multivariable analysis. All non-residential human movement metrics were highly colinear (absolute correlation coefficient range 0·82–0·94) with change in mobility in grocery or pharmacy showing lower, but still high, collinearity (0·82–0·86). No public health and social measures, nor human movement behaviours, had strong correlation with any environmental variables.

**Figure 3:**
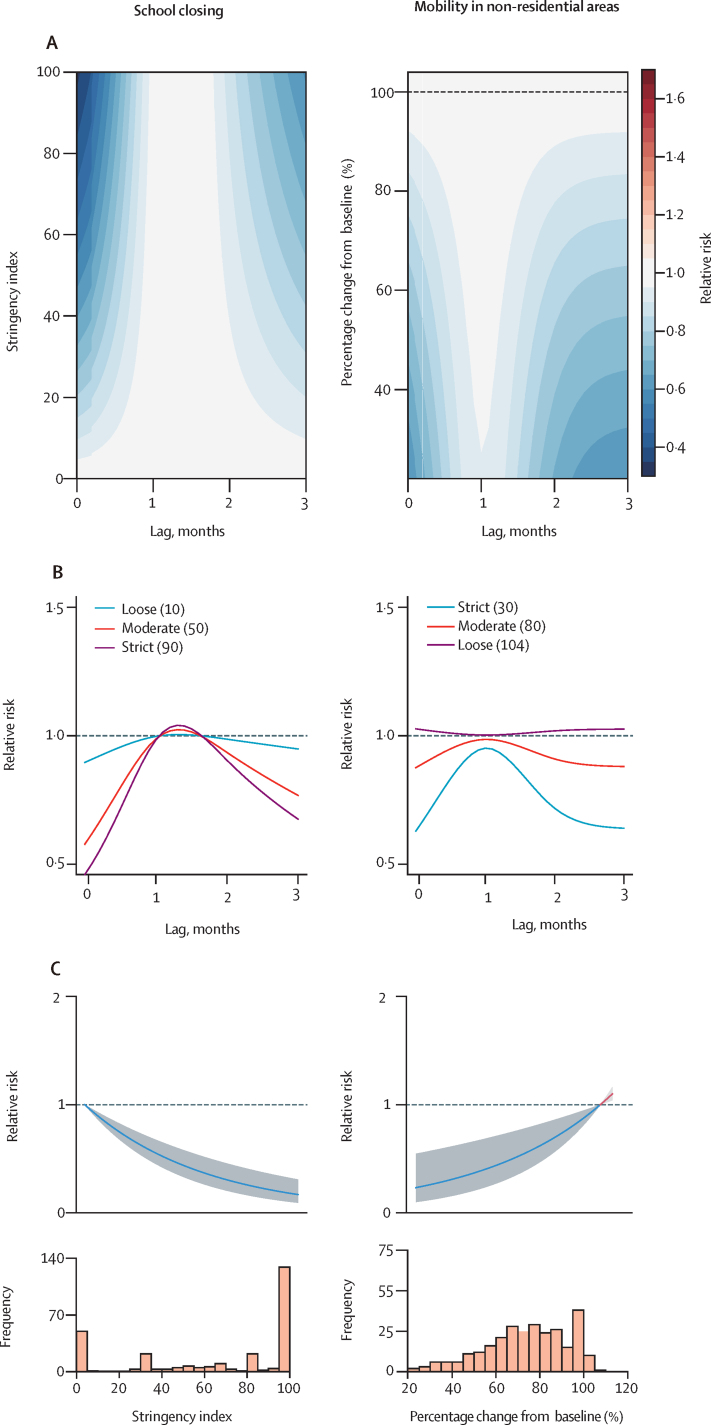
Association between different selected intervention and human movement variables with dengue risk over different lags The index for public health and social measures ranges from 0 to 100. A higher score indicates a more stringent, more geographically comprehensive COVID-19 response policy (0 for no response policy and 100 for the most stringent response policy). The baseline of human mobility was the median for the first 5 weeks of 2020 (Jan 3–Feb 6), which was defined as 100%. (A) Contour plot of the association between selected intervention and human movement variables and risk of dengue, relative to the baseline, without government interventions (ie, 0 for public health and social measures and 100 for human movement behaviours). The deeper the shade of red, the greater the increase in relative risk of dengue compared with the baseline. The deeper the shade of blue, the greater the decrease in relative risk of dengue compared with the baseline. (B) Dengue lag–response association for loose, moderate, and strict government interventions relative to the baseline. (C) Cumulative lags over the 4-month time periods associations between public health and social measures or human movement behaviours and risk of dengue, relative to the baseline, without government interventions. Shaded regions are 95% CIs. Predictions are from the intervention models. Cumulative lags over the 4-month time periods are shown in the appendix (p 10).

After covariate selection, the human movement behaviours model retained all non-residential variables except change in workplace, whereas the public health and social measures model only retained school closing (figure 2). Consistent with the univariable analysis, these variables were negatively associated with dengue risk (RR range 0·01–0·17), but the magnitude of association varied over different lag periods (Figure 2, Figure 3). School closing was associated with the biggest decrease in dengue risk at short lags (0–1 month) and—to a lesser extent—long lags (3 month; figure 3). To reduce the impact of collinearity, we aggregated human movement behaviour variables (arithmetic mean) into residential and non-residential. Only the non-residential variable was retained by the human movement behaviour model, and a positive association with dengue risk was identified (figure 3). Low values of non-residential movement showed the strongest protective effects at short (0–1 month) and medium to long (2–3 months) time lags.

The selected variables belonged to highly colinear clusters. This means that we cannot accurately rule out an association between dengue risk and restrictions on gathering size or cancelling public events. This analysis does, however, suggest that there is relatively less evidence for an association between dengue risk and stay at home requirements, closure of public transport, restrictions on domestic and international movement, and workplace closures.

By comparing observed and predicted cases (via the historical model) between April and December, 2020, we estimate that 0·72 million (95% CI 0·12–1·47) fewer dengue cases occurred (table), representing a 35% (9–56) decrease that is potentially attributable to COVID-19-related disruption.

**Table:** The number of cases observed and predicted (April–Dec, 2020) after the implementation of public health and social measures

		**Observed cases in 2020**	**Predicted cases in 2020, n (95% CI)**	**Averted cases, n (95% CI)**	Percentage of averted cases*
Latin America		851 933	727 359 (438 058–1 161 136)	−124 574 (−413 875 to 309 203)	−29% (−94 to 27)
	Belize	1350	1198 (304–2553)	−152 (−1046 to 1203)	−77% (−344 to 47)
	Bolivia	12 086	15 403 (2819–38 365)	3317 (−9267 to 26 279)	−42% (−329 to 68)
	Brazil	586 945	162 972 (13 799–528 274)	−423 973 (−573 146 to −58 671)	−1225% (−4154 to −11)
	Colombia	35 456	84 116 (23 885–173 679)	48 660 (−11 571 to 138 223)	39% (−48 to 80)
	Costa Rica	7760	9142 (2516–19 419)	1382 (−5244 to 11 659)	−26% (−208 to 60)
	Dominican Republic	931	19 519 (5311–40 896)	18 588 (4380 to 39965)	93% (82 to 98)
	Ecuador	8882	27 862 (6703–62 513)	18 980 (−2179 to 53 631)	49% (−33 to 86)
	El Salvador	3048	16 674 (4509–36 938)	13 626 (1461 to 33 890)	73% (32 to 92)
	Guatemala	2580	30 900 (7792–65 772)	28320 (5212 to 63192)	87% (67 to 96)
	Honduras	13 978	17 515 (5040–35 915)	3537 (−8938 to 21 937)	−18% (−177 to 61)
	Jamaica	224	8040 (1732–18 950)	7816 (1508 to 18 726)	95% (87 to 99)
	Mexico	99 504	215 028 (47 650–503 367)	115 524 (−51 854 to 403 863)	24% (−109 to 80)
	Nicaragua	33 685	16 085 (4375–34 811)	−17 600 (−29 310 to 1126)	−208% (−670 to 3)
	Panama	1146	9038 (2627–19 171)	7892 (1481 to 18 025)	81% (56 to 94)
	Peru	40 509	32 175 (7526–67 705)	−8334 (−32 983 to 27 196)	−101% (−438 to 40)
	Venezuela	3849	61 693 (15 386–127 462)	57 844 (11 537 to 123 613)	90% (75 to 97)
Southeast Asia		299 291	1 147 357 (641 700–1 766 550)	848 066 (342 409 to 1 467 259)	71% (53 to 83)
	Cambodia	10 085	84 745 (21 717–186 668)	74 660 (11 632 to 176 583)	82% (54 to 95)
	Laos	6863	18 300 (4799–41 347)	11 437 (−2064 to 34 484)	42% (−43 to 83)
	Malaysia	54 932	114 854 (27 976–246 123)	59 922 (−26 956 to 191 191)	24% (−96 to 78)
	Philippines	35 838	377 556 (95 003–819 952)	341 718 (59 165 to 784 114)	85% (62 to 96)
	Singapore	30 370	21 653 (5531–46 632)	−8717 (−24 839 to 16 262)	−121% (−449 to 35)
	Thailand	62 134	287 687 (71 906–614 232)	225 553 (9772 to 552 098)	66% (14 to 90)
	Vietnam	99 069	242 561 (59 857–543 930)	143 492 (−39 212 to 444 861)	34% (−66 to 82)
Total		1 151 224	1 874 716 (1 266 555–2 618 688)	723 492 (115 331 to 1 467 464)	35% (9 to 56)

Data are based on the historical model projected on 2020 environmental and epidemiological conditions.

* Percentage averted cases = averted cases / predicted cases.

This reduction was more pronounced in countries in southeast Asia than in Latin America (table). In southeast Asia all countries except Singapore were predicted to have substantial reductions in dengue cases with the largest reductions seen in the Philippines and Cambodia. In Latin America, most (nine of 16) countries had fewer cases than expected; however, Belize, Bolivia, Brazil, Costa Rica, Honduras, Nicaragua, and Peru experienced more cases than anticipated. Brazil, in particular, remains a major outlier that negatively skews regional and global estimates of the percentage of averted cases, accounting for 51% of all observed dengue cases between April and December, 2020. This discrepancy between expected and observed cases in 2020 in Brazil might be related to the less stringent public health and social measures, variable adherence,^26^ and more modest changes in human movement behaviours that occurred in the country in 2020 (appendix pp 17–18).

**Figure 4:**
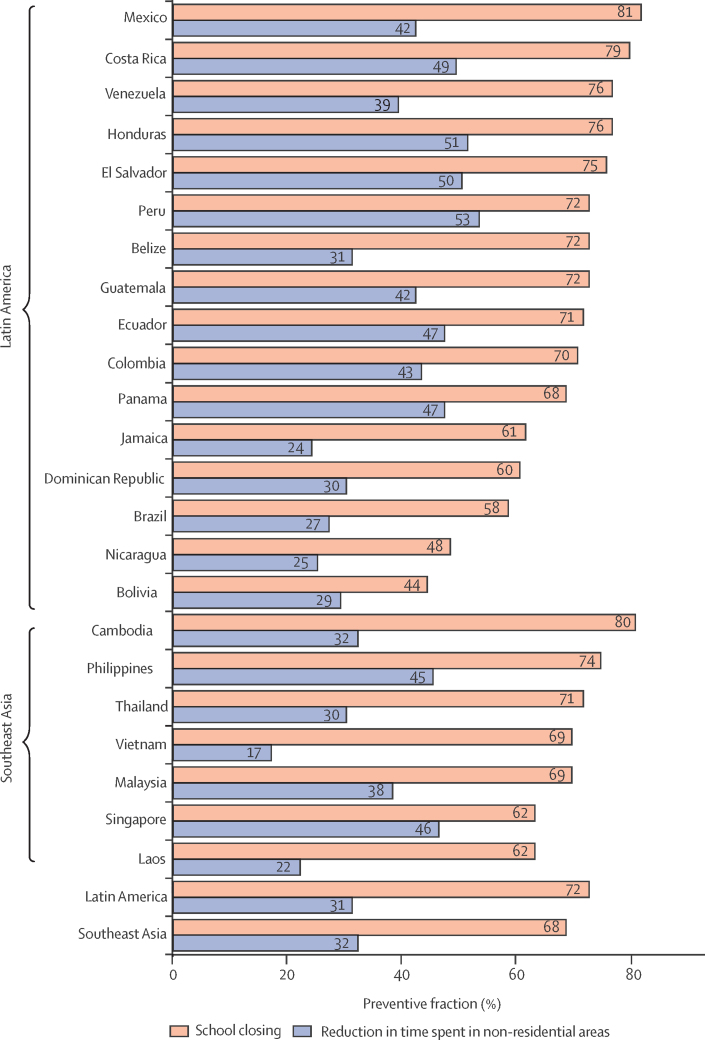
Preventive fraction of dengue cases averted in 2020 attributable to specific public health and social measures and human movement behaviours, by region and country Preventive fractions were calculated using the intervention models.

We then tested what proportion of the difference between expected (historical model) and observed case counts in April to December, 2020 could be explained by the specific public health and social measures and human movement behaviour variables in our analysis (appendix p 10). School closures in the public health and social measures model explained 70·95% (95% CI 55·55–80·48) of the reduction, whereas reductions in movement in non-residential locations in the human movement behaviour model explained 30·95% (15·57–43·65; appendix p 30). Even in countries with low or negative estimates of averted cases (table), such as Brazil, variation in monthly case counts could be explained by the public health and social measures models (figure 4), suggesting these countries would have experienced lower dengue case counts if public health and social measures had been more stringent or declines in non-residential movement been more substantial.

## Discussion

By combining the most globally comprehensive collection of dengue and COVID-19 response data, we show that the sudden decline in dengue cases in April, 2020 is associated with the imposition of restrictions and changes in human movement behaviours. We show that school closures and declines in non-residential trips have the strongest association with reduced dengue risk. Combined, we estimate that 0·72 million (95% CI 0·12–1·47) fewer dengue cases occurred in 2020 than would have occurred in the absence of COVID-19-related disruption.

It remains to be seen how many of these 0·72 million cases are truly averted or just delayed until later years as pre-COVID-19 human movement behaviours re-establish. By using distributed lag non-linear models, we were able to show that public health and social measures and human movement behaviours confer both short-term (0–1 month) and medium to long-term (2–3 months) protective effects. Continued observation and re-analysis will be needed to assess longer term effects. Disruption to routine vector control (eg, household larval inspections and community clear-up campaigns) could suffer long-term effects that are not observable until the next dengue epidemic.^7^ In the long term, more routine measurement of population seroprevalence for dengue^27^ and a better understanding of how treatment-seeking behaviour changes at different phases of dengue and COVID-19 epidemics (as access to care and rapid diagnostics changes) will be important to interpret changes in reported caseloads. Continued monitoring of dengue trends in 2021 and beyond will be key, including the continued collection of human movement data, better data on adherence to public health and social measures,^26^ and the use of disease forecasting systems to detect and respond to dengue epidemics when they do occur.

Theoretically, COVID-19-related disruption could increase or decrease dengue transmission through mechanisms such as mosquito control disruption, reduced human movement restricting geographical spread, and reduced time spent in high risk non-residential environments.^7^ These hypothetical changes in risk would probably act over different timescales, with reducing time in high-risk environments leading to the most immediate reductions, whereas restricting spread and disruption to mosquito control could take 1–3 months to have substantial effects. This mixture of effects might explain why we estimate varying levels of protection at different lags.

Although we caution against overinterpretation of the selection, magnitude, lag, or direction of specific variables in our analysis, some consistent trends could guide further studies. Reductions in non-residential movement and closure of schools had the strongest evidence of an association with reduced dengue risk among the variables analysed. Understanding where dengue transmission occurs in different settings (eg, home, workplace, or school) remains a major knowledge gap. Targeting mosquito control measures to households of individuals with dengue has long been recommended and practiced^2^ under the assumption that mosquito exposure within, or in close proximity to, the home drives transmission. Despite this, household cluster studies rarely identify strong clustering of transmission around houses,^28^ and a competing theory has emerged that transmission occurs in shared spaces away from the home or is driven by the movement of people that allows the virus to expand into new pockets of human susceptibility.^29^, ^30^ By showing that dengue risk is more closely associated with reduced time spent in public areas, we add evidence to this theory. Our findings imply that schools and other commonly visited public areas (or travel between home and these places) could be dengue transmission hotspots. These findings are consistent with the apparent concentration of symptomatic cases in children younger than 15 years^31^ and the main vector of dengue, *Aedes aegypti*, preferentially biting during the day. If supported by further outbreak investigation studies, this finding would suggest a greater emphasis is needed on dengue control in public places, and in schools in particular. These findings might also be of relevance to the dynamics of other arboviral diseases (eg, Zika and chikungunya) and to infectious diseases more generally, and serves as an example that COVID-19-related disruption does not always result in adverse effects.

Our findings have several limitations. First, owing to data availability, we did not include information on dengue serotypes or genotypes, which are well known drivers of dengue outbreaks.^32^ Such switches might explain outliers, such as Singapore, where a sustained switch in the predominant serotype from DENV-1 or DENV-2 to DENV-3 could have led to the observed increases in incidence in 2020.^32^ Second, explicitly considering the timing of changes in public health and social measures and human movement behaviours relative to the usual dengue season might also have improved model fit. ^33^

Third, we were unable to control for potential changes in dengue reporting that might have occurred due to COVID-19 disruption. By showing that case fatality rates in 2020 were not abnormal, we provide evidence against the theory that reduced dengue incidence in 2020 is due solely to underreporting. Some countries, such as Sri Lanka, have also reported undertaking additional community outreach activities for dengue during the COVID-19 pandemic to restrict the effects of any reduced treatment-seeking behaviours.^34^ Effects on case presentation, diagnosis, and reporting are likely to be complex, country-specific, and delayed. Additionally, if dengue cases were substantially underreported then we would expect a rapid rise in reported cases as COVID-19 restrictions are lifted, as opposed to a more gradual rise due to resurgence of dengue transmission. Despite countries in Asia relaxing domestic COVID-19 restrictions in late 2020, we did not observe rapid rises in reported dengue cases. A more detailed temporal analysis of fatal and non-fatal cases of multiple acute conditions would give more insight into how disease surveillance has changed during the COVID-19 pandemic.

Fourth, we were not able to include all countries seriously affected by dengue in our analysis because publicly available monthly case reports could not be found for some countries. Indonesia reports the highest number of dengue cases in southeast Asia and, with equatorial seasonality, would have improved our historical and intervention models. However, the reported annual case decline in Indonesia (138 127 in 2019 and 108 303 in 2020)^35^ is within the range of other countries in the region and is unlikely to change our main findings.

Fifth, in this study we use penalised complexity priors for estimating parameters using integrated nested laplace approximation. Penalised complexity priors are well suited for penalising more complex models with multiple variables; however, model fit and predictions of the number of averted cases might differ with different prior specifications.

Lastly, our analysis was restricted to national-scale dengue and movement dynamics. There is probably substantial sub-national heterogeneity in the size and strength of association between movement restrictions and dengue risk that will be important to quantify. One priority for research is measuring how this association varies between urban and rural areas, with urban areas typically having much higher baseline movement than rural areas.

In summary, this study is the most geographically comprehensive study to date to show that the substantial reduction in dengue cases seen in 2020 is potentially attributable to COVID-19-related disruption. Although it remains unknown what effect these restrictions will have on dengue dynamics in the long term, the unique circumstances of the COVID-19 pandemic might give new insights into the development and targeting of new and existing interventions for dengue.

## Data sharing

All data and code used in this analysis are fully documented and freely available from the following GitHub repository: https://github.com/huaiyutian/Dengue_COVID-19

## Declaration of interests

AWS works as a consultant to WHO. All other authors declare no competing interests.
