# Supplementary material for: Measuring the effects of COVID-19-related disruption on dengue transmission in southeast Asia and Latin America: a statistical modelling study
Source: Lancet Infect Dis. 2022 May;22(5):657–67. doi: 10.1016/S1473-3099(22)00025-1 (PMC8890758; doi:10.1016/S1473-3099(22)00025-1)
Supplement: Supplementary appendix [file mmc1.pdf]

THE LANCET
Infectious Diseases

Supplementary appendix

This appendix formed part of the original submission and has been peer reviewed. We post it as supplied by the authors.

Supplement to: Chen Y, Li N, Lourenço J, et al. Measuring the effects of COVID-19-related disruption on dengue transmission in southeast Asia and Latin America: a statistical modelling study. *Lancet Infect Dis* 2022; published online March 2. https://doi.org/10.1016/S1473-3099(22)00025-1.

Content

1. Additional information on data collection 2
2. Detailed information on the analytical framework 2
2.1 Computation on Mann-Kendall test 2
2.2 Details on the Spatiotemporal Bayesian hierarchical model 3
2.3 Details on the DLNM 4
2.4 Computation of the preventive fraction 6
2.5 Model variable description table 6
1. Supplementary Table S1-S4 8
2. Supplementary Figure S1-S20 13
References 36

1. Additional information on data collection

Dengue. The incidence of dengue fever is calculated by dividing the number of dengue cases by the total population that year. The dengue incidence in 2020 is subtracted from the average of the incidence in the same time from 2014 to 2019 in the corresponding country and then divided by the average of the incidence to obtain the net change of dengue incidence in 2020.
2m temperature. The temperature of the air 2 meters above the earth's surface. Taking atmospheric conditions into account, 2m temperature is calculated using the interpolation method between the lowest model layer and the surface. The Kelvin temperature minus 273.15 is converted to degrees Celsius (°C).
Surface temperature. The temperature of the earth's surface. Surface temperature is the theoretical temperature required to satisfy the surface energy balance. It represents the temperature at the top layer, which has no heat capacity and therefore responds instantly to changes in surface flux. The Kelvin temperature minus 273.15 is converted to degrees Celsius (°C).
Relative humidity. The humidity of the air 2 meters above the surface. Its original data is the dew point temperature of 2 meters. Combined with temperature and pressure, it can be used to calculate the relative humidity.
Total precipitation. Liquid and frozen water, including rain and snow, falling on the earth's surface. It is the sum of mass precipitation (precipitation produced by large-scale weather patterns, such as troughs and cold fronts) and convective precipitation (precipitation produced by convection when the air in the lower part of the atmosphere is warmer and less dense than the air in the upper part of the atmosphere). The precipitation variable does not include fog, dew, or precipitation that evaporates in the atmosphere before it falls to the Earth's surface. Precipitation is measured at a depth of meters.
Convective precipitation. The cumulative amount of convective precipitation falling to the Earth's surface. It is the precipitation phenomenon caused by atmospheric convection movement, and the rainfall is large in a short period of time. The units of precipitation are depth in metres.
Government stringency index (SI). SI is the simple average of 9 component indicators, including 8 indexes of containment and closure policies and 1 public information campaigns of health system policies. OxCGRT published the calculation formula and detailed definition of specific indicators.[1] The indexes range from 0 to 100. A higher score indicates a more stringent COVID-19 response policy (0 for no response policy and 100 for the most stringent response policy).
Human mobility. The Google Mobility Trends data set measures the number of visitors per day in a particular site category and compares this change to the baseline day before the outbreak of the COVID-19 pandemic. The baseline is the median value of the first five-week period of 2020 (3 January to 6 February). The baseline human mobility was defined as 100% in this study.

2. Detailed information on the analytical framework

2.1 Computation on Mann-Kendall test

In the Mann - Kendall test, the null hypothesis H_0 for climate time series data

$(X_1,...,X_{12})$ were independent of n, random variable with the distribution of the sample. The alternative hypothesis H_1 is bilateral inspection, to $k,j \le 12$, and $k \ne n$, X_i and X_j distributed is not the same, the test statistic S calculation is as follows:

$$S = \sum_{k=1}^{n-1} \sum_{j=k+1}^{n} Sgn(X_j - X_{12})$$

S is a normal distribution with a mean of 0 and a variance of $Var(S) = n(n-1)(2n+5)/18$.

When $n > 10$, the standard positive system variable is calculated by the following formula:

$$Z \begin{cases} \dfrac{s-1}{\sqrt{\operatorname{var}(s)}} & S > 0 \\ 0 & S = 0 \\ \dfrac{s+1}{\sqrt{\operatorname{var}(s)}} & S < 0 \end{cases}$$

So, in the trend of the bilateral inspection, on a given a confidence level α, if $|Z| \ge Z_{(1-\sigma/2)}$, the null hypothesis is not acceptable. On a confidence level, the time series data exist obvious up or down trend. For statistic Z, when it is greater than zero, it has an upward trend, and when it is less than zero, it has a downward trend. The absolute value of Z in greater than or equal to 1.28, 1, 64 and 2.32, respectively by the reliability of 90%, 95%, 99% of the test of significance.

2.2 Details on the Spatiotemporal Bayesian hierarchical model

We specified a spatiotemporal Bayesian hierarchical model that responded to the monthly dengue cases in 23 countries from 2014 to 2019.[2] Hypothesis of negative binomial distribution explains the possible overdistribution of dengue cases:

$$y_{c,t} | dengue\ case_{c,t}, \kappa \sim NegBin(dengue\ case_{c,t}, \kappa)$$

$$\log(dengue\ case_{c,t}) = NS(climate\ factors_{c,t}, var\ df, lag\ df) + \beta_{c,m(t)} + \varphi_{c,a(t)} + \upsilon_{c,a(t)}$$
$$+ annual\ anomaly_{(c,a(t)-1)} + GDP_{c,a(t)} + offset(population_{c,a(t)})$$

Where, $y_{c,t}$ is the number of dengue cases, and is equal to $a_{(t)} = 2014,...,2019$, the number of annual population per 100,000 $population_{c,a(t)}$ multiplied by the estimated value of country $c = 1,...,23$ for the incidence of unknown dengue fever $dengue\ case_{c,t}$, κ is an over-discrete parameter. At the same time, we also test the Poisson distribution model, but the goodness of fit standard, including the deviance information criterion (DIC) is higher, so we use the negative binomial formula to consider residual over-discreteness.

We first construct a baseline model that includes spatiotemporal random effects to explain inter-annual variability in seasonal and spatiotemporal correlation structures at national levels. Using cyclic first-order random walk priors, which allow each month to depend on the previous month. Interannual variations and long-term trends are explained by annual spatial random effects, which make any annual trend different over

the entire time period at a given location. Unstructured random effects allow for independent region-specific noise, such as differences in media ecology, healthcare access, and reporting rates. Structured spatial random effects allow for dependence between adjacent countries due to common environmental and socio-economic characteristics such as climatic zones and mobility of people.

The model parameters are estimated using the integrated nested Laplace approximation in the Bayesian framework. INLA is directly used for full Bayesian inference in disease mapping, avoiding the computationally intensive Markov chain Monte Carlo technique.[3] Parametric uncertainty is resolved by assigning prior distributions to parameters. Month autocorrelation random effects were used for 23 countries, in which each month's effect was derived from the effect of the immediately preceding month. This country-specific monthly random effect, $\beta_{c,m(t)}$ was assigned a cyclic random walk of order one, or first difference prior distribution, in which each effect is derived from the immediately preceding effect,

$$\beta_{c,m(t)} - \beta_{c,m(t)-1} \sim N(0, \sigma^2_{\beta})$$

where $\beta_{c,m(1)}$ represents the parameter estimate for the month of January for country c.

Fig.S13 shows the marginal posterior distribution of the monthly random effects in each country using the best-fit "historical" model. Spatial unstructured and structured random effects with specific countries can account for inter-annual variations due to unknown and unmeasurable spatial characteristics (e.g., the introduction of new dengue serotypes or arboviruses at specific times and places) and long-term trends. For the spatial components, we use a modified Besag-York-Mollie (BYM) model with a scaled spatial component, which helps to assign interpretive superpriors and make these superpriors transferable across different geographic environments. The modified BYM model consists of one precision parameter and one mixing parameter. The precision parameter represents the marginal precision and controls the variability explained by the spatial effect. The mixing parameter distributes existing variables between an unstructured and structured component, $\varphi_{c,a(t)} + \upsilon_{c,a(t)}$. The unstructured component helps explain unknown or unobserved confounders, such as population immunity, quality of health care, and local vector control interventions. In addition to the single-scale parameters in the negative binomial model, it introduces an additional source of variability (potential effects) into the model, which helps to model excessive dispersion. Structured components assume that if regions share boundaries, there is spatial dependency that acts as a substitute for spatial autocorrelation between nearby regions due to shared environmental or socio-economic characteristics.[4]

2.3 Details on the DLNM

The influence of meteorological factors on human health is nonlinear (such as J, V or U shaped relationship) and shows lagged effect. Gasparrini introduced the Distributed Lag non-linear model (DLNM) into the study of the health effects of air temperature for the first time.[5,6] Based on the ideas of traditional models such as generalized linear model and generalized additive model, they introduced a cross basis process to describe

the distribution of dependent variables in the independent variable dimension and lag dimension at the same time, so as to evaluate the lag effect and nonlinear effect of exposure factors at the same time.

We used a distributed lag nonlinear model (DLNM) to establish a negative binomial distribution to derive an estimate of a specific variable-dengue cases association, expressed as relative risk (RR). This model can describe complex nonlinearity and lag correlation by defining a combination of two functions that define the traditional exposure - response correlation and the additional lag - response correlation, respectively. Lag-response associations represent temporal changes in given exposure risk and estimates the distribution of immediate and delayed effects that accumulate during the lag. The technical details and terminology are explained in detail in literature and tutorials.[6]

Basis function refers to the function that converts the independent variable into a new variable set and includes it in the design matrix of the model to estimate its effect.

The dependent variable is the number of dengue cases.[6] In this study, the exposure factors were 2m temperature, convective precipitation environmental factors and PHSM policy factors. For environmental covariates, we used population-weighted averages to reduce the biases particular for sparsely populated countries with big meteorological gradients:

$$PrEnv_c = \frac{\sum_{i=1}^{n} pop_i \times Env_i}{\sum_{i-1}^{n} pop_i}$$

Where $PrDen_c$ is the population-weighted environmental factors in country c, pop_i is the population in pixel i, and Env_i is the environmental factor in pixel i, and n is the total number of pixels in country c.

The new variable generated by the transformation of the basis function of the independent variable is called the change relationship between the basis variable and the dependent variable can be described in a more detailed way through the transformation of the independent variable, and a more accurate exposure response relationship can be obtained. The basis functions of the independent variable dimension include the meteorological cross basis function of a natural cubic spline of time with 3 degrees of freedom (df), and the cross basis function of PHSM and human mobility of a natural cubic spline of time with 1 df. Their boundary sections are the minimum and maximum values of their respective variables, respectively.

Due to the hysteresis of the influence of exposure, the outcome of the day may be affected by the exposure L months ago at the longest. In order to describe the hysteresis effect of exposure, it is also necessary to select a basis function to transform the hysteresis to form a matrix. Based on the available literature and knowledge of the vector-borne process of human disease transmission, the lag time for weather variables is up to three months.[7-9] In order to describe the lag effect of exposure, the basis function of the lag dimension is a natural cubic spline of time with 1 df, and the maximum lag is 3 months. The boundary nodes are placed at 0 and 3 months respectively, and two internal nodes are distributed for 1 month and 2 months. The difference between cases reported in last year and mean annual cases 2013-2019 were introduce to account for population immunity built up. Since dengue risk and vulnerability to COVID-19 related

disruptions is likely to be affected by wealth, GDP per capita was also included in the model. Population size was included as an offset variable. Q AIC information criteria are used to evaluate the model.

2.4 Computation of the preventive fraction

Measuring attributable risk and prevention is an integral part of epidemiological analysis, especially when it is aimed at planning and evaluating public health interventions. Forward attribution is well suited to separating the risks attributable to different ranges of components because their sum matches the overall risk. In addition, from current exposures to future risks, a forward-looking view seems better suited to quantifying the health burden and prevention from a specific exposure event, as it is based on more consistent counterfactual conditions.[7]

We select the forward attribution method to quantify the preventive fraction (PF) for COVID-19 dengue transmission. For a particular month t in 2020, the dengue prevention attributable to a policy index or human mobility X_t is defined as the fraction $PF_{x,t}$ of dengue cases with 3 months as the maximum lag period, defined by:

$$PF_{x,t} = 1 - \exp(\sum_{l=0}^{L} \beta_{xt}, 1)$$

With $\sum \beta_{xt,1}$ as the overall cumulative log-relative risk for policy index or human mobility x_t in month *t*. The risk estimate $\sum \beta_{xt,1}$ is calculated by the cumulative correlation of the policy index or human mobility over three months and re-centered on the base values of the non-PHSM. The basic value is the counterfactual condition to define the culpable protective factors. Therefore, the attributable prevention can be interpreted as the excess cases due to non-basic value.
This method is described in detail in Gasparrini's previous article.[7]

2.5 Model variable description table

**Variable**	**Description**
$c = 1,...,23$	Number of countries
$t = 1,...,72$	Number of months between January 2014 and December 2019
$a_{(t)} = 2014,...,2019$	Annual
$m_{(t)} = 1,...,12$	January to December
κ	Overdispersion parameter
NS	Natural cubic spline
var df	Degree of freedom of variable
lag df	Degree of freedom of lag

$y_{c,t}$	Number of reported cases
$annual\ anomaly_{(a(t)-1)}$	Short-term immunity
$\beta_{c,m(t)}$	Country-specific monthly random effect
$\varphi_{c,a(t)} + \upsilon_{c,a(t)}$	Yearly spatial random effects.
Climate factors	Surface temperature and convective precipitation
Population	Per 100 000 population in each country
PHSM	Public health and social measures
HMB	Human movement behaviours
$\hat{y}$	The mean predicted case counts for 2020 from the historical model on the log scale

1. Supplementary Table S1-S4

Table S1: **2014-2019 Monthly Dengue - Environment (i.e., non-PHSM, non-HMB) covariate DLNM model adequacy results for models of increasing complexity.** The deviance information criterion (DIC) and the cross-validated (CV) mean logarithmic score for models of increasing complexity. Lower scores indicate a better fitting model.

Model	Dengue incidence rate estimate	DIC	CV mean log score
Baseline	Environmental covariates + Spatiotemporal random effects	27345.71	8.288
mt	Base model + mt DLNM	27302.61	8.27
st	Base model + st DLNM	27301.43	8.269
rh	Base model + rt DLNM	27320.72	8.275
tp	Base model + tp DLNM	27335.83	8.28
cp	Base model + cp DLNM	27328.32	8.275
mt+rh	Base model + (mt + rh) DLNM	27276.04	8.259
mt+tp	Base model + (mt + tp) DLNM	27279.92	8.26
mt+cp	Base model + (mt + cp) DLNM	27274.22	8.256
st+rh	Base model + (st + rh) DLNM	27275.17	8.259
st+tp	Base model + (st + tp) DLNM	27275.15	8.258
**st+cp**	**Base model + (st + cp) DLNM**	**27269.41**	**8.255**
rh+tp	Base model + (rh + tp) DLNM	27323.98	8.274
rh+cp	Base model + (rh + cp) DLNM	27312.84	8.268
mt+rh+tp	Base model + (mt + rh + tp) DLNM	27278.59	8.259
mt+rh+cp	Base model + (mt + rh + cp) DLNM	27275.31	8.256
st+rh+tp	Base model + (st + rh + tp) DLNM	27275.33	8.258
st+rh+cp	Base model + (st + rh + cp) DLNM	27270.91	8.255

Environmental covariates: population immunity;
mt: 2m temperature; st: Surface temperature; rh: Relative humidity; tp: Total precipitation; cp: Convective precipitation.

Table S2: **2014-2019 Monthly Dengue - Environment (i.e., non-PHSM, non-HMB) covariate DLNM model including per capita GDP adequacy results for models of increasing complexity.** The deviance information criterion (DIC) and the cross-validated (CV) mean logarithmic score for models of increasing complexity. Lower scores indicate a better fitting model.

Model	Dengue incidence rate estimate	DIC	CV mean log score
Baseline+GDP	Environmental covariates + Spatiotemporal random effects	27344.83	8.287
mt+GDP	Base model + mt DLNM	27302.15	8.27
st+GDP	Base model + st DLNM	27301.64	8.269
rh+GDP	Base model + rt DLNM	27320.67	8.275
tp+GDP	Base model + tp DLNM	27336.96	8.28
cp+GDP	Base model + cp DLNM	27329.10	8.276
mt+rh+GDP	Base model + (mt + rh) DLNM	27275.46	8.259
mt+tp+GDP	Base model + (mt + tp) DLNM	27279.23	8.26
mt+cp+GDP	Base model + (mt + cp) DLNM	27274.69	8.256
st+rh+GDP	Base model + (st + rh) DLNM	27275.22	8.259
st+tp+GDP	Base model + (st + tp) DLNM	27275.34	8.258
**st+cp+GDP**	**Base model + (st + cp) DLNM**	**27269.45**	**8.254**
rh+tp+GDP	Base model + (rh + tp) DLNM	27324.94	8.275
rh+cp+GDP	Base model + (rh + cp) DLNM	27312.86	8.268
mt+rh+tp+GDP	Base model + (mt + rh + tp) DLNM	27278.65	8.259
mt+rh+cp+GDP	Base model + (mt + rh + cp) DLNM	27274.75	8.256
st+rh+tp+GDP	Base model + (st + rh + tp) DLNM	27274.08	8.257
st+rh+cp+GDP	Base model + (st + rh + cp) DLNM	27271.13	8.255

Environmental covariates: population immunity + GDP per capita;
mt: 2m temperature; st: Surface temperature; rh: Relative humidity; tp: Total precipitation; cp: Convective precipitation.

Table S3: **Overall cumulative 4-month association between PHSM, HMB and dengue transmission relative risk.** 95% CI = 95% confidence interval.

CHI	PHSM (%)		HMB (%)				
	SI (95% CI)	C1 (95% CI)	Non-Residential (95% CI)	Transit (95% CI)	Park (95% CI)	Grocery (95% CI)	Retail (95% CI)
0	1	1	/	/	/	/	/
10	0.86 (0.8 - 0.93)	0.84 (0.79 - 0.89)	/	/	/	/	/
20	0.74 (0.63 - 0.87)	0.7 (0.62 - 0.79)	/	0.03 (0 - 0.23)	/	/	0.03 (0 - 0.25)
30	0.64 (0.5 - 0.81)	0.59 (0.49 - 0.7)	0.28 (0.13 - 0.59)	0.05 (0.01 - 0.28)	0.05 (0.01 - 0.31)	0.01 (0 - 0.04)	0.05 (0.01 - 0.3)
40	0.55 (0.4 - 0.76)	0.49 (0.38 - 0.62)	0.33 (0.17 - 0.63)	0.08 (0.02 - 0.33)	0.08 (0.02 - 0.36)	0.01 (0 - 0.07)	0.08 (0.02 - 0.36)
50	0.48 (0.32 - 0.71)	0.41 (0.3 - 0.56)	0.4 (0.23 - 0.68)	0.12 (0.03 - 0.4)	0.12 (0.03 - 0.43)	0.03 (0.01 - 0.1)	0.12 (0.03 - 0.42)
60	0.41 (0.25 - 0.66)	0.34 (0.24 - 0.49)	0.48 (0.31 - 0.74)	0.18 (0.07 - 0.48)	0.19 (0.07 - 0.51)	0.06 (0.02 - 0.16)	0.18 (0.07 - 0.5)
70	0.35 (0.2 - 0.62)	0.29 (0.19 - 0.44)	0.58 (0.42 - 0.8)	0.28 (0.13 - 0.58)	0.28 (0.13 - 0.6)	0.12 (0.06 - 0.26)	0.28 (0.13 - 0.6)
80	0.31 (0.16 - 0.58)	0.24 (0.15 - 0.39)	0.69 (0.56 - 0.86)	0.43 (0.26 - 0.69)	0.43 (0.26 - 0.71)	0.25 (0.15 - 0.4)	0.43 (0.26 - 0.71)
90	0.26 (0.13 - 0.54)	0.2 (0.12 - 0.35)	0.83 (0.75 - 0.93)	0.65 (0.51 - 0.83)	0.66 (0.51 - 0.84)	0.5 (0.39 - 0.64)	0.65 (0.51 - 0.84)
100	0.23 (0.1 - 0.51)	0.17 (0.09 - 0.31)	1	1	1	1	1
110	/	/	/	/	1.52 (1.18 - 1.96)	2.01 (1.57 - 2.58)	/
120	/	/	/	/	/	4.06 (2.47 - 6.65)	/

C1-School closing; HMB-Reduction in human mobility; '/'- Without the intervention; RR of the baseline values of PHSM=0 and Mobility=100 is 1.

***Table S4:* Overall cumulative proportion of dengue cases averted by PHSM and HMB in each countries and region.**

Areas	PHSM (%)		HMB (%)				
	SI (95% CI)	C1 (95% CI)	Non-Residential (95% CI)	Transit (95% CI)	Park (95% CI)	Grocery (95% CI)	Retail (95% CI)
All	54.18 (32.76 - 69.13)	70.95 (55.55 - 80.48)	30.95 (15.75 - 43.65)	75.37 (44.21 - 90.91)	70.51 (41.41 - 87.44)	29.48 (12.17 - 49.03)	75.56 (44.89 - 91.15)
Americas	53.6 (33.27 - 69.93)	71.53 (58.11 - 81.42)	30.66 (15.75 - 45.38)	73.54 (43.45 - 89.04)	71.25 (41.51 - 87.11)	29.05 (12.54 - 50.04)	75.55 (45.02 - 90.55)
Southeast Asia	56.46 (36.39 - 70.94)	67.73 (53.38 - 78.03)	32.1 (16.64 - 47.15)	81.08 (53.28 - 93.79)	66.56 (37.13 - 83.6)	31.5 (13.76 - 51.1)	75.64 (43.14 - 91.92)
Belize	51.75 (31.44 - 67.71)	71.88 (57.55 - 82.1)	30.66 (15.87 - 45.23)	82.7 (51.05 - 94.44)	54.34 (25.4 - 77.34)	38.99 (18.2 - 61.94)	69.73 (35.9 - 88.99)
Bolivia	31.04 (16.21 - 47.52)	44.27 (30.84 - 57.34)	28.81 (12.45 - 45.28)	76.86 (42.03 - 93.55)	70.58 (32.2 - 91.59)	59.31 (26.96 - 84.8)	77.19 (36.29 - 94.12)
Brazil	46.38 (25.21 - 63.3)	57.91 (42.66 - 68.71)	27.41 (14.34 - 40.03)	69.86 (42.56 - 85.98)	71.64 (42.73 - 87.83)	14.92 (6.98 - 23.29)	74.9 (43.91 - 89.68)
Colombia	55.37 (33.69 - 71.37)	69.69 (54.23 - 80.08)	43.26 (23.24 - 60.57)	85.61 (57.54 - 96.23)	81.28 (50.33 - 94.1)	63.49 (35.7 - 82.69)	86.6 (57.61 - 96.4)
Costa Rica	60.59 (39.89 - 75.46)	79.43 (67.16 - 87.24)	49.14 (27.74 - 64.82)	87.37 (65.3 - 96.03)	87.02 (63.37 - 95.92)	60.4 (35.7 - 76.39)	84.5 (59.4 - 94.94)
DR	45.05 (23.94 - 62.4)	60.11 (44.73 - 72.92)	30.19 (15.92 - 45.37)	80.03 (48.73 - 94.45)	63.43 (31.18 - 84.33)	44.69 (22 - 65.73)	71.47 (39.5 - 90.08)
Ecuador	56.35 (33.67 - 72.99)	70.86 (56.64 - 81.22)	47.15 (24.43 - 64.37)	87.31 (59.09 - 96.36)	83.72 (54.21 - 95.26)	69.2 (37.76 - 87.82)	86.27 (56.76 - 96.44)
ES	63.84 (39.82 - 78.75)	75.17 (61.89 - 84.36)	50.25 (30.94 - 66.95)	88.73 (62.92 - 97.1)	85.7 (57.62 - 95.93)	69.49 (42.19 - 86.35)	88.91 (63.47 - 96.95)
Guatemala	61.6 (39.68 - 77.7)	71.74 (58.04 - 81.98)	42.42 (21.31 - 59.1)	88.26 (61.97 - 96.81)	74.33 (44.47 - 89.17)	60.77 (33.72 - 78.69)	81.05 (51.1 - 93.65)
Honduras	65.33 (39.23 - 80.26)	75.5 (63.64 - 84.9)	50.8 (28.25 - 66.31)	90.92 (67.44 - 97.8)	77.98 (49.59 - 90.7)	73.46 (43.15 - 88.86)	89.65 (60.87 - 97.57)
Jamaica	47.18 (28.92 - 63.72)	61.15 (45.21 - 72.96)	24.45 (12.51 - 36.21)	57.64 (31.23 - 77.43)	63.23 (34.84 - 83.51)	36.6 (17.91 - 53.89)	59.14 (31.91 - 78.82)
Mexico	62.47 (41.39 - 76.74)	80.53 (68.91 - 87.96)	42.12 (23.62 - 56.82)	84.17 (60.96 - 94.18)	78.02 (50.89 - 90.6)	35.67 (19.18 - 49.41)	80.92 (55.87 - 93.04)
Nicaragua	17.67 (10.46 - 24.72)	47.72 (36.15 - 58.61)	25.08 (13.48 - 35.86)	64.6 (39.72 - 80.35)	50.37 (27.4 - 66.84)	31.1 (16.52 - 43.74)	60.87 (35.27 - 77.45)
Panama	52.71 (30.22 - 69.71)	67.61 (52.13 - 78.44)	46.6 (24.19 - 65.17)	87.79 (59.7 - 96.49)	84.22 (50.1 - 95.92)	69.57 (39.35 - 87.62)	88.5 (59.73 - 97.21)
Peru	63.29 (40.49 - 77.26)	72.11 (58.74 - 81.01)	52.78 (29.61 - 70.39)	90.28 (66.05 - 97.36)	84.71 (53.8 - 95.47)	73.31 (43.77 - 88.69)	90.63 (66.71 - 97.83)
Venezuela	61.66 (38.19 - 76.13)	75.88 (61.91 - 84.76)	39.29 (19.9 - 55.09)	81.64 (54.11 - 93.05)	74.24 (47.27 - 88.4)	52.61 (29.17 - 70.38)	79.38 (49.02 - 91.81)

Cambodia	46.54 (28.51 - 60.75)	79.68 (67.18 - 87.31)	31.73 (17.08 - 43.58)	83.47 (60.77 - 93.46)	44.8 (25.36 - 60.14)	37.9 (20.9 - 53)	57.61 (34.21 - 74.03)
Laos	50.39 (29.12 - 66)	61.73 (47.71 - 72.53)	22.06 (11.99 - 32.5)	75.6 (46.96 - 89.94)	28.07 (14.98 - 39.57)	28.89 (12.27 - 46.78)	60.86 (28.41 - 81.09)
Malaysia	52.43 (31.54 - 66.74)	68.61 (54.06 - 78.75)	38.24 (19.45 - 53.67)	85.35 (56.52 - 95.53)	71.64 (43.26 - 89.38)	44.79 (21.13 - 65.4)	84.65 (55.09 - 96)
Philippines	59.48 (36.64 - 74.35)	73.58 (60 - 83.04)	45.07 (24.45 - 62.68)	89.92 (67.51 - 97.47)	70.77 (40.97 - 86.72)	61.32 (33.37 - 81.22)	87.88 (59.57 - 96.99)
Singapore	56.42 (37.27 - 70.09)	61.78 (48.19 - 73.33)	45.63 (26.63 - 61.58)	86.75 (62.57 - 95.9)	83.25 (55.51 - 94.62)	31.78 (16.35 - 44.88)	84.43 (57.96 - 94.98)
Thailand	57.24 (36.84 - 72.11)	71.22 (57.3 - 80.5)	30.47 (16.53 - 43.08)	80 (55.49 - 92.02)	68.4 (41.06 - 84.67)	9.55 (3.79 - 16.57)	64.32 (36.53 - 82.42)
Vietnam	62.02 (41.83 - 75.74)	69.1 (55.56 - 78.64)	17.11 (9.16 - 25.16)	54.59 (28.57 - 75.77)	56.03 (33.07 - 71.65)	8.37 (1.62 - 19.83)	57.65 (29.59 - 76.84)

DR, Dominican Republic; ES, El Salvador; *HMB-Reduction in human mobility; '/'- Without the intervention.*

2. Supplementary Figure S1-S20

Figure S1: **Seasonal variation in monthly dengue incidence for 2020 and average monthly dengue incidence for 2014-2019 in Latin America and Southeast Asia.** (A) Seasonal variation of monthly dengue incidence for countries in Latin America. (B) Seasonal variation of monthly dengue incidence for countries in Southeast Asia. The percentage is the relative change ratio of annual dengue incidence in 2020 to the mean of 2014-2019. The gray dotted line is when countries started taking COVID-19 PHSM.

A

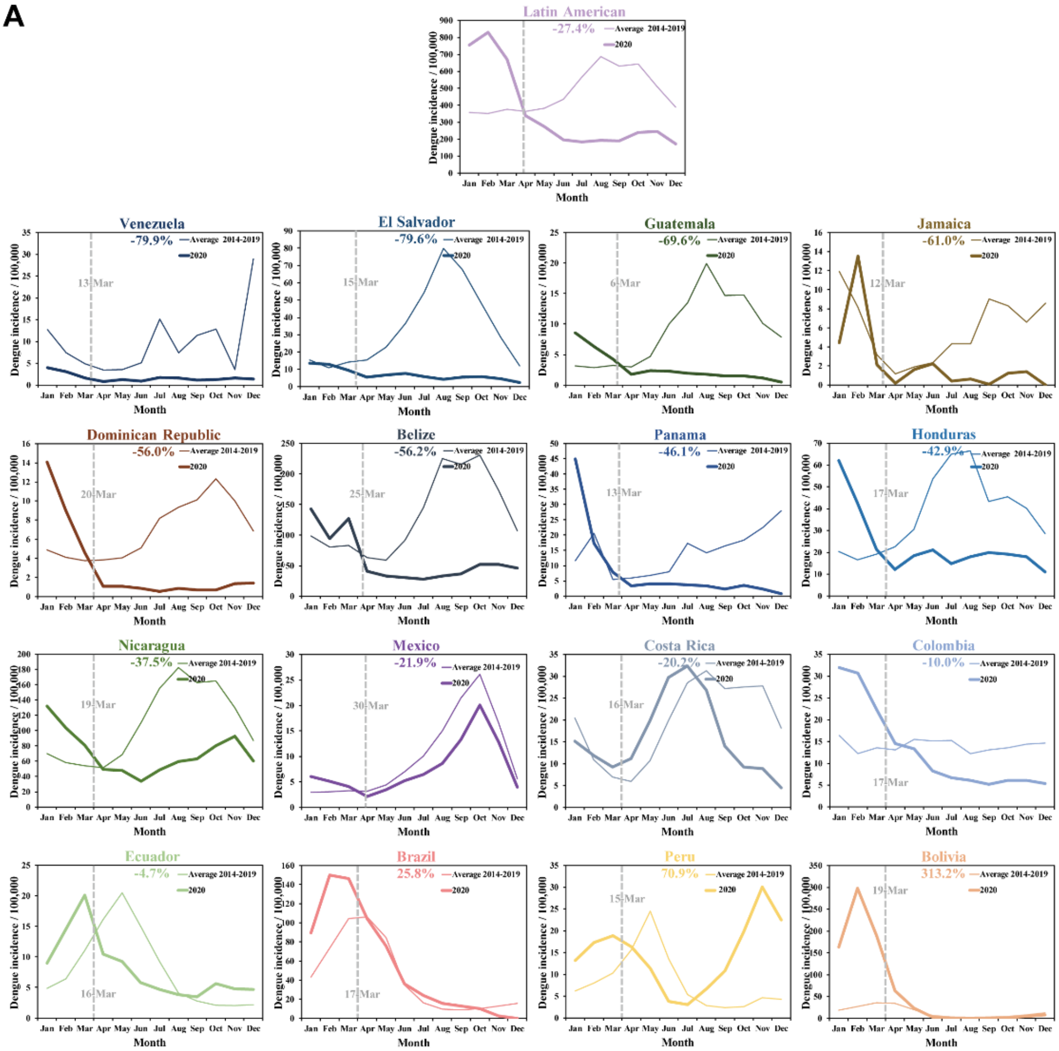

B

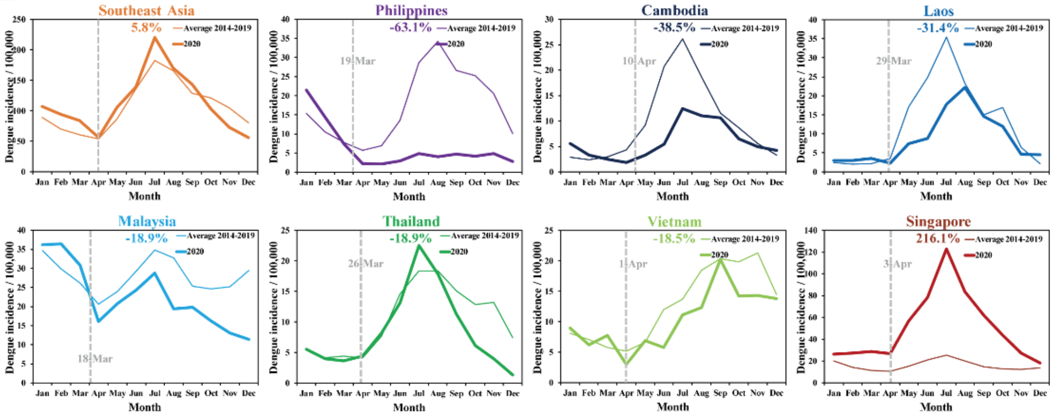

Figure S2: **Seasonal variation in monthly dengue incidence for 2020 and maximum monthly dengue incidence for 2014-2019 in Latin America and Southeast Asia.** (A) Seasonal variation of monthly dengue incidence for countries in Latin America. (B) Seasonal variation of monthly dengue incidence for countries in Southeast Asia. The percentage is the relative change ratio of annual dengue incidence in 2020 to the maximum value of 2014-2019. The gray dotted line is when countries started taking COVID-19 PHSM.

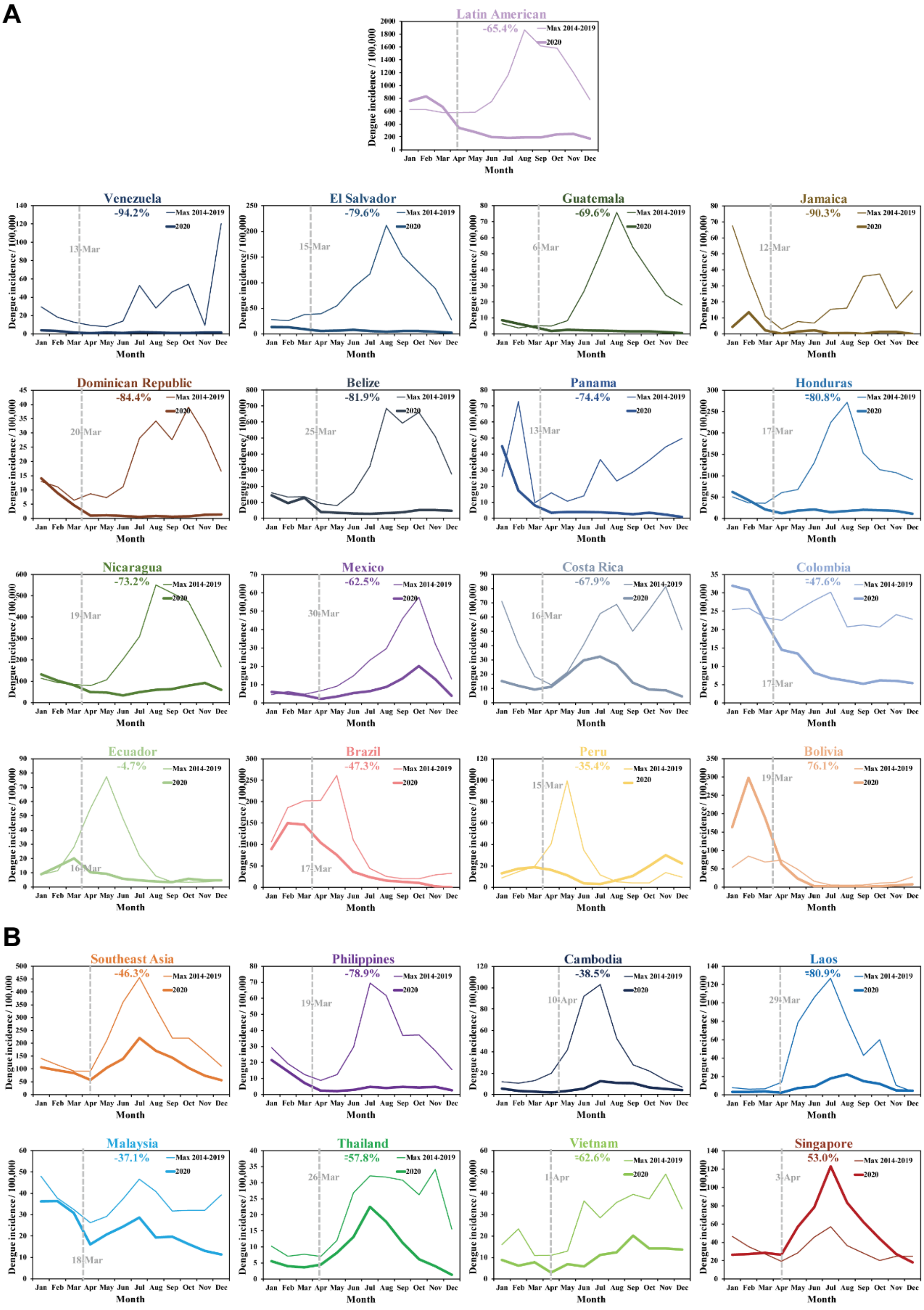

***Figure S3:* Yearly dengue case fatality rates from 2014-2019.**

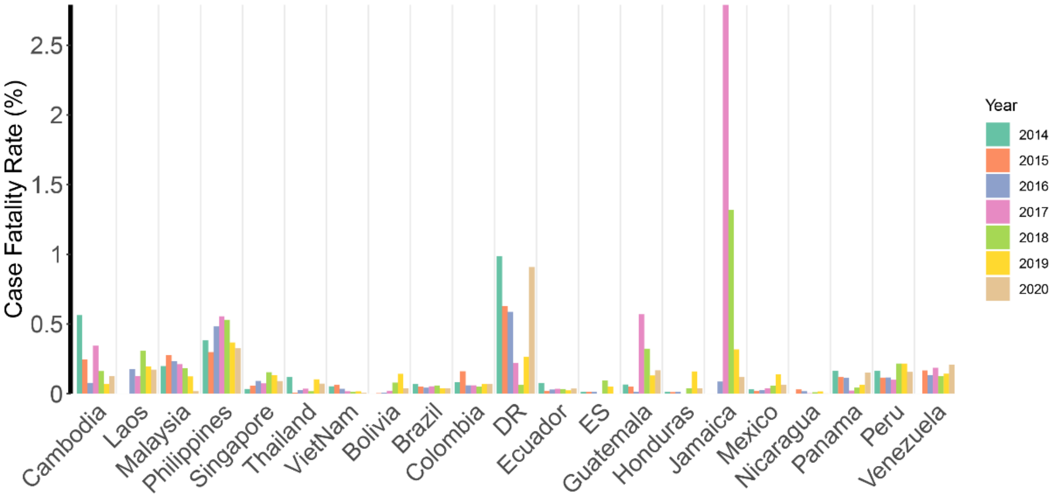

DR, Dominican Republic; ES, El Salvador.

Figure S4: **Distribution of annual dengue incidence for each country in 2019 and 2020.**

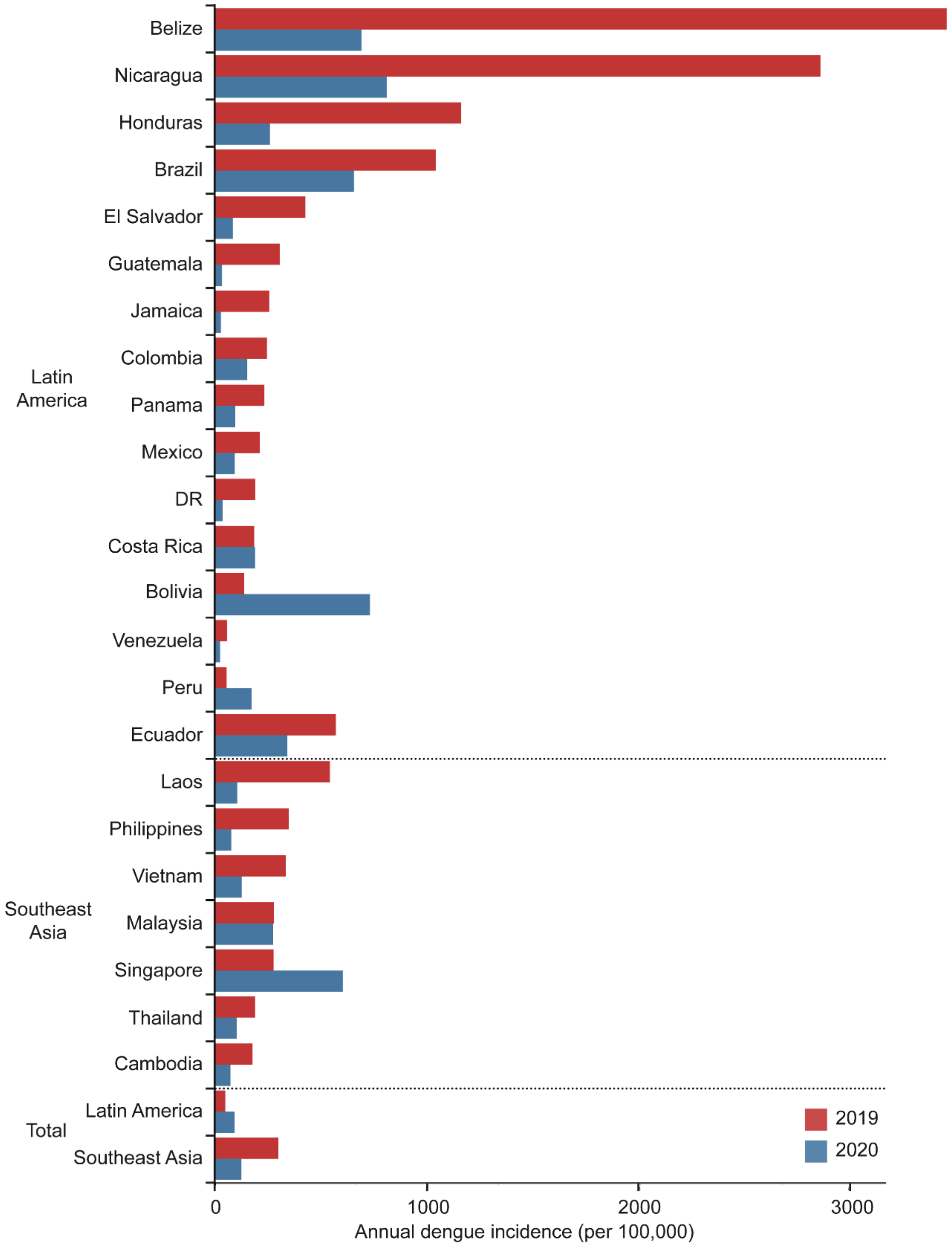

***Figure S5:* The change of 8 PHSM index response to COVID-19 in the Latin America and Southeast Asia, 2020.** When the baseline value is 0, there is no COVID-19 response policy. The data are percentages.

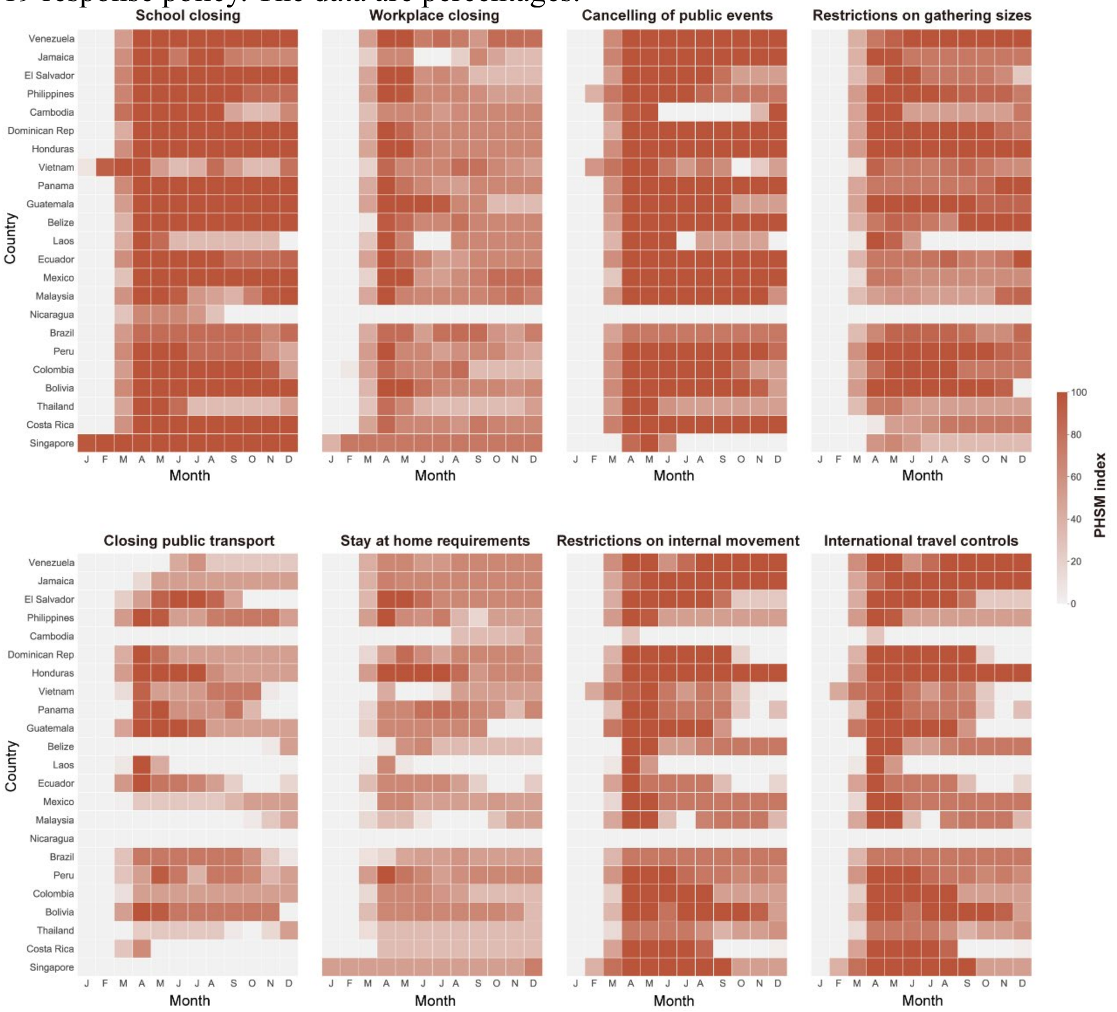

Figure S6: **The change of HMB response to COVID-19 in the Latin America and Southeast Asia, 2020.** There are six categories of community mobility: residential, workplace, transit stations, park, grocery and pharmacy, retail and recreation. Global human mobility data is obtained from Google Community Mobility Reports. The selected observation time period is the mean value within 35 days after the national emergency response. The baseline is the median value, for the corresponding day of the week, during January 3, 2020 to February 6, 2020.The baseline value is 100 and data are percentages.

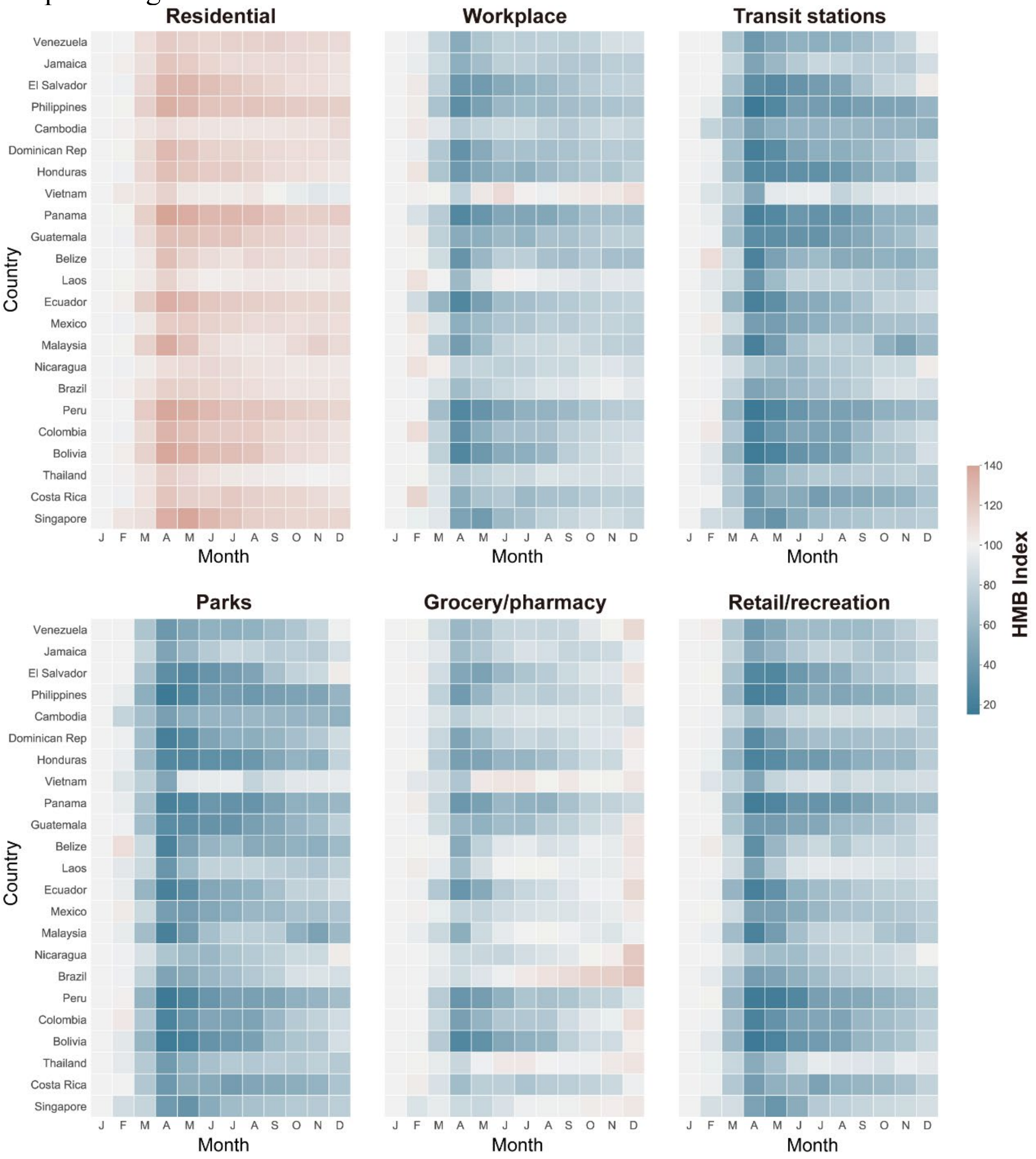

Figure S7: **Mann-Kendall test for significance of trends in meteorological factor for each country in the Latin America and Southeast Asia, 2014-2020.** (A) Mann-Kendall significance test for surface temperature. (B) Mann-Kendall significance test for convective precipitation. The red line represents the UF value, the blue line represents the UB value, and the dashed line represents the 95% CI. When the red line exceeds the critical line of I, the variable has a significant change trend at this time.

A **Skin temperature**

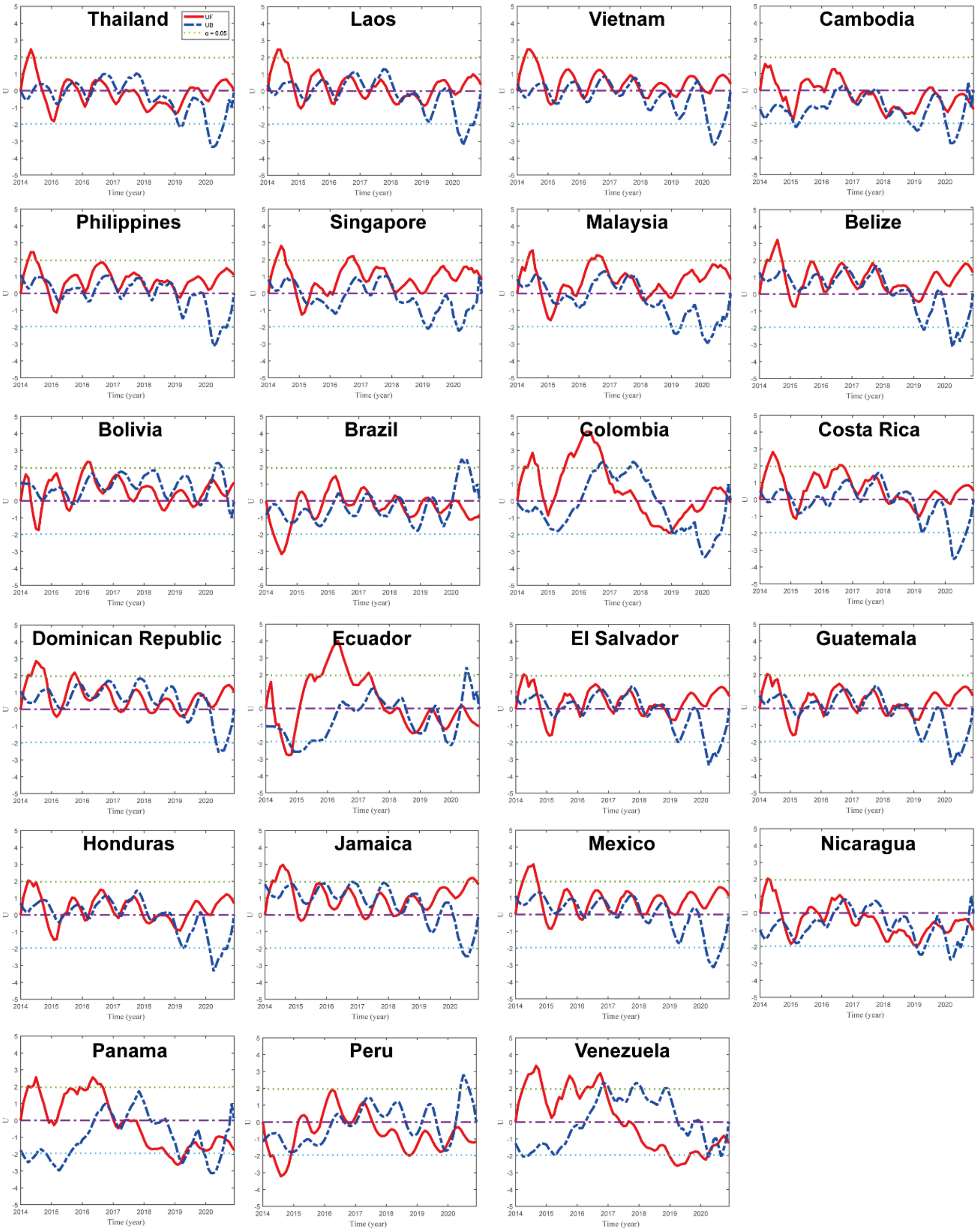

B Convective precipitation

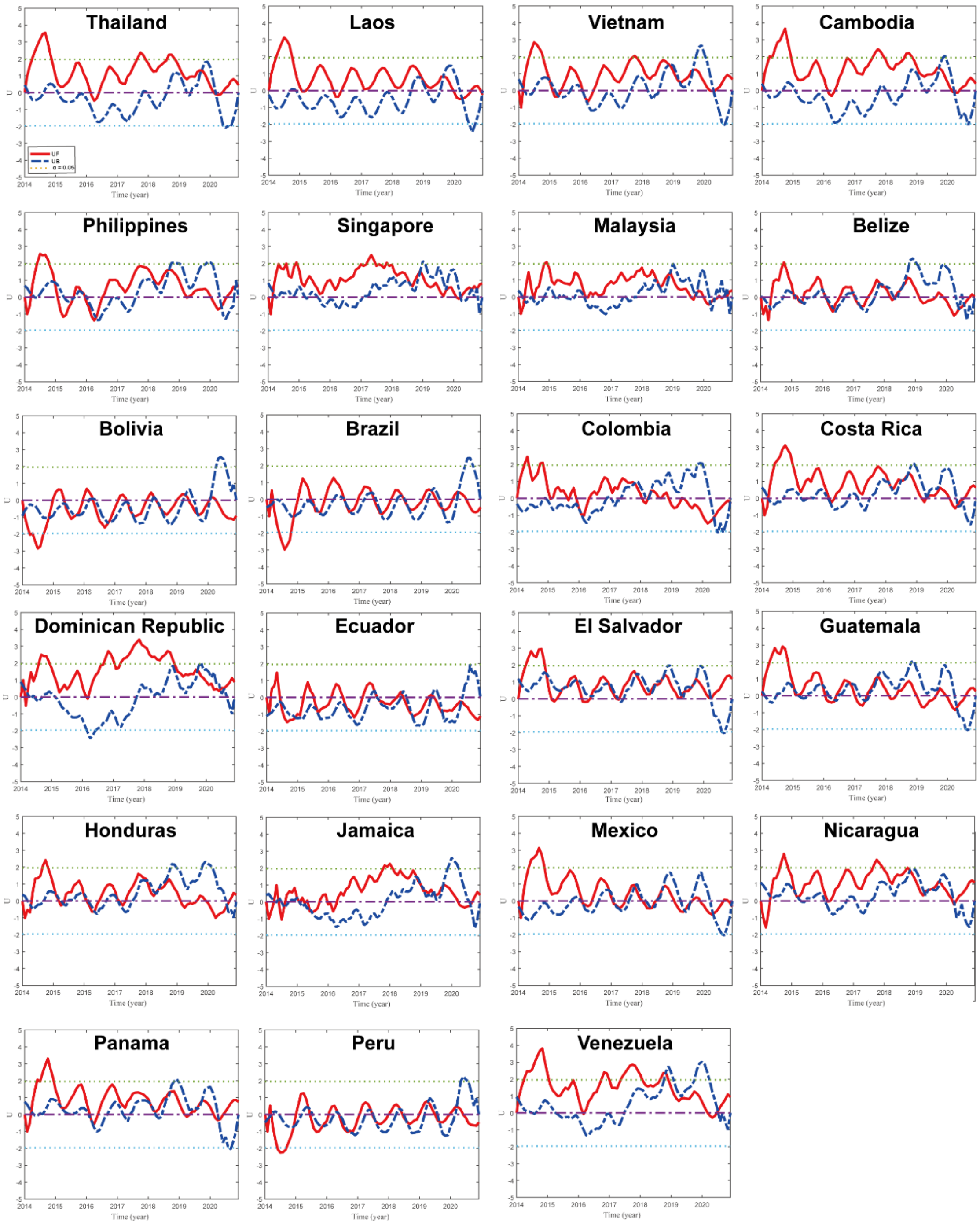

***Figure S8:* Posterior distributions of country-specific autocorrelated monthly random effects.** Posterior mean of marginal posterior distribution of the autocorrelated month random effects (e.g., the annual cycle) at the linear predictor scale from January to December for the 23 countries in Latin America and Southeast Asia. This shows the contribution of the random effects to the log of the dengue incidence rate (DIR) using the "historical" model.

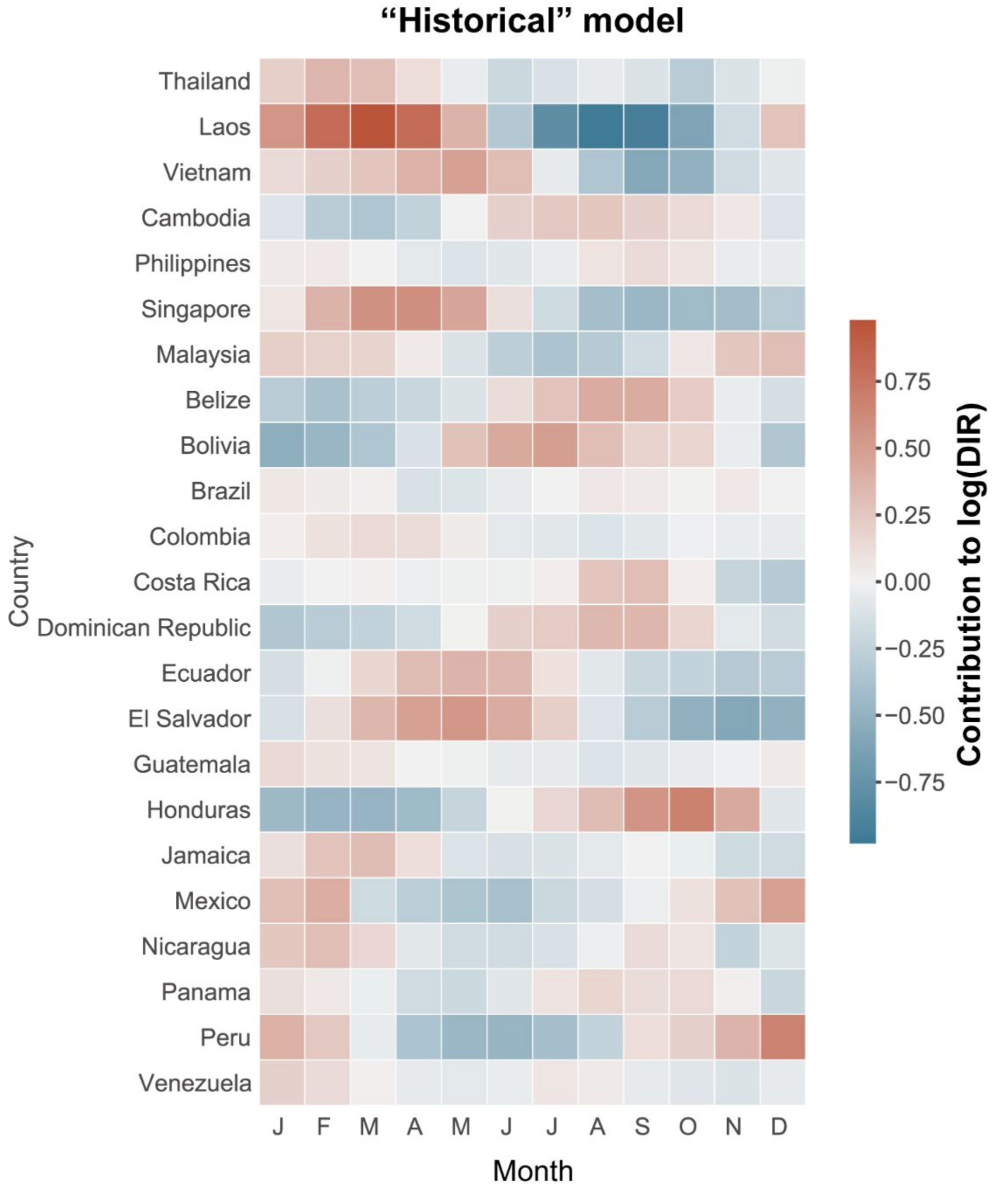

Figure S9: **Contribution of year-specific spatial random effects to dengue incidence rate estimates.** Marginal posterior mean of the combined spatially structured and unstructured random effects at the linear predictor scale in 2020. This shows the contribution of the spatial random effects to the log of the dengue incidence rate using the "historical" model.

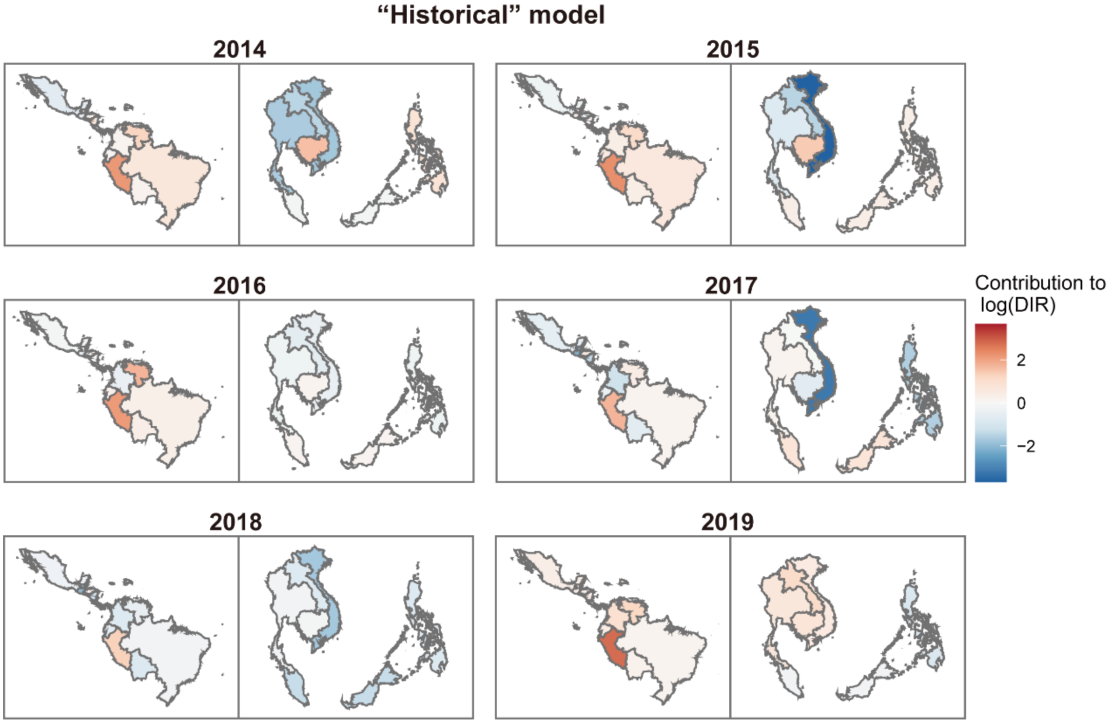

Figure S10: **Added value of using "historical" model compared to baseline model.** Difference between mean absolute error (MAE) for the baseline model (state-specific monthly random effects and year-specific spatial random effects) and MAE for the "historical" model. Countries with positive values (red) suggest that capturing the nonlinear and delayed impacts of climate factors, improves the model in these areas. Countries with negative values (blue) suggest that climate factors information did not improve the model fit and other unexplained factors may dominate space-time dynamics in these countries. The MAE of the "Historical" model was smaller than the baseline model for 12 of the 23 countries (52%).

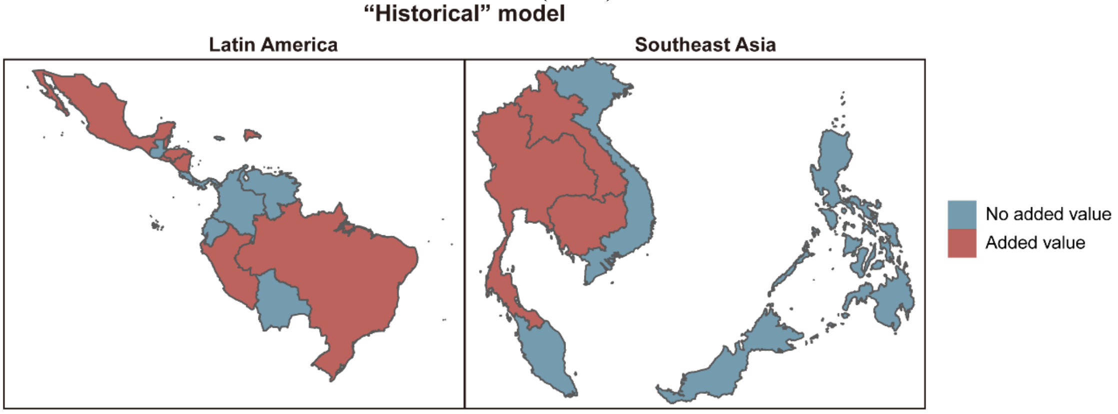

Figure S11: **Sensitivity testing of "historical" models using complete and seperate (Latin American and Southeast Asian) datasets.** Countries with positive MAE values (red) indicate that modeling independently of countries in two large regions can improve these regional models. Countries with negative values (blue) indicate that modeling independently for two large regions does not improve the model fit, and is better for all countries. In 12 of the 23 countries (52%), the MAE of the complete dataset model was smaller than that of the two-region dataset model.

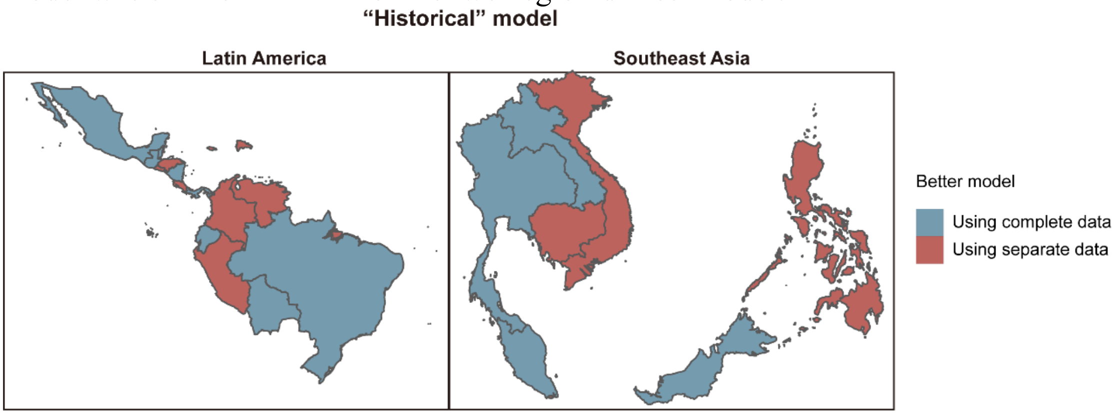

Figure S12: **The association between different selected intervention variables with dengue risk over different lags.** (A) Contour plot of the association between PHSM and risk of dengue, relative to the baseline without government interventions (PHSM = 0). The deeper the shade of red, the greater the increase in relative risk (RR) of dengue compared with the baseline. The deeper the shade of blue, the greater the decrease in RR of dengue compared with the baseline. (B) Dengue lag–response association for loose (PHSM = 10), moderate (PHSM = 50), and strict (PHSM = 90) government interventions relative to the baseline. (C) Cumulative lags over the three month associations between PHSM and risk of dengue, relative to the baseline without government interventions. Shaded regions mark the prediction with 95% empirical CI. Predictions are from the "intervention" models. The PHSM index ranges from 0 to 100. A higher score indicates a more stringent, more geographically comprehensive COVID-19 response policy (0 for no response policy and 100 for the most stringent response policy).

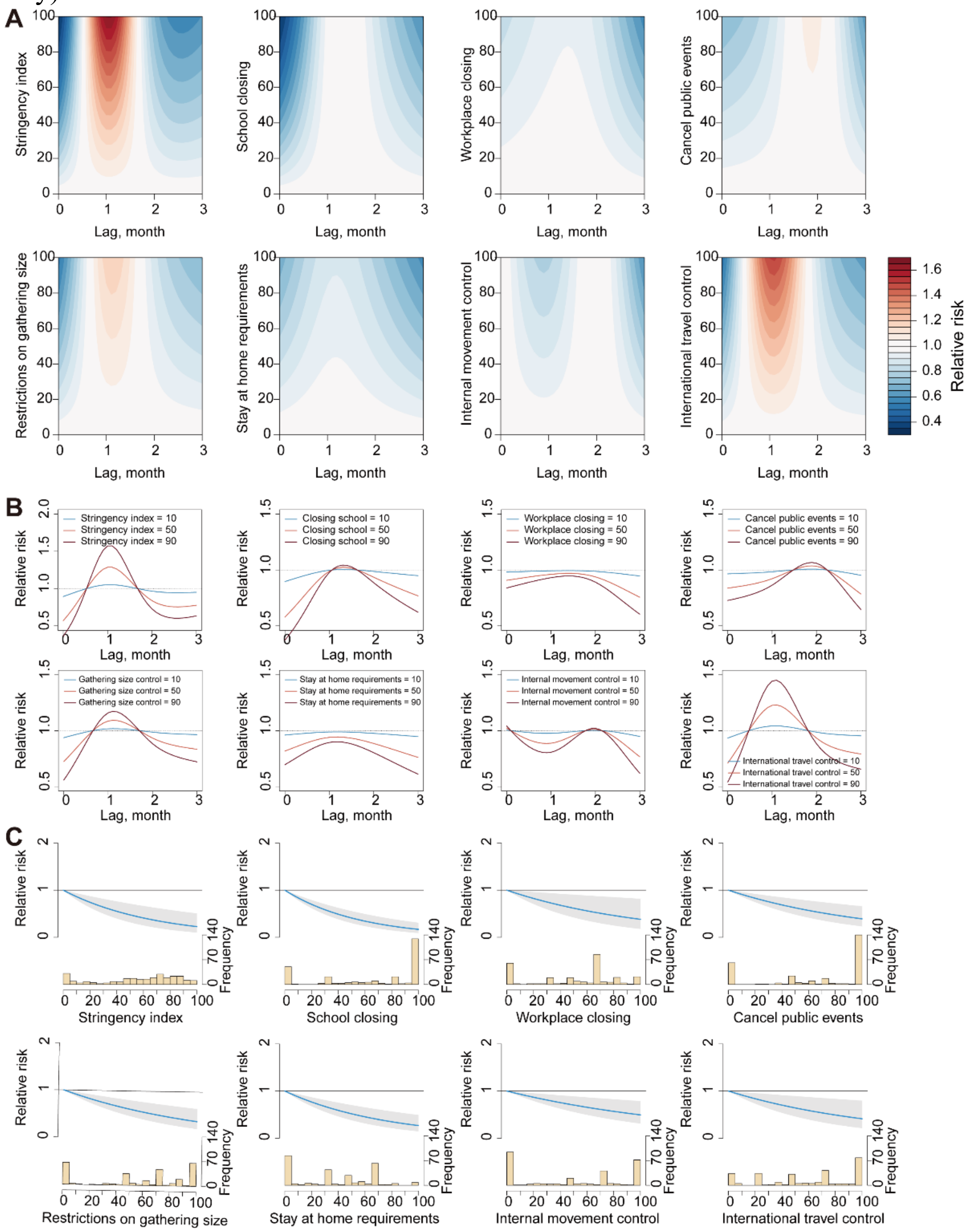

***Figure S13:* The association between different selected human movement variables with dengue risk over different lags.** (A) Contour plot of the association between HMB and risk of dengue, relative to the baseline without government interventions (HMB = 100). The deeper the shade of red, the greater the increase in relative risk (RR) of dengue compared with the baseline. The deeper the shade of blue, the greater the decrease in RR of dengue compared with the baseline. (B) Dengue lag–response association for loose (HMB = max), moderate (HMB = 80), and strict (HMB = 30) government interventions relative to the baseline. (C) Cumulative lags over the three month associations between HMB and risk of dengue, relative to the baseline without government interventions. Shaded regions mark the prediction with 95% empirical CI. Predictions are from the "intervention" models. The baseline of human mobility was the median for the first five weeks of 2020 (3 January to 6 February), which was defined as 100%.

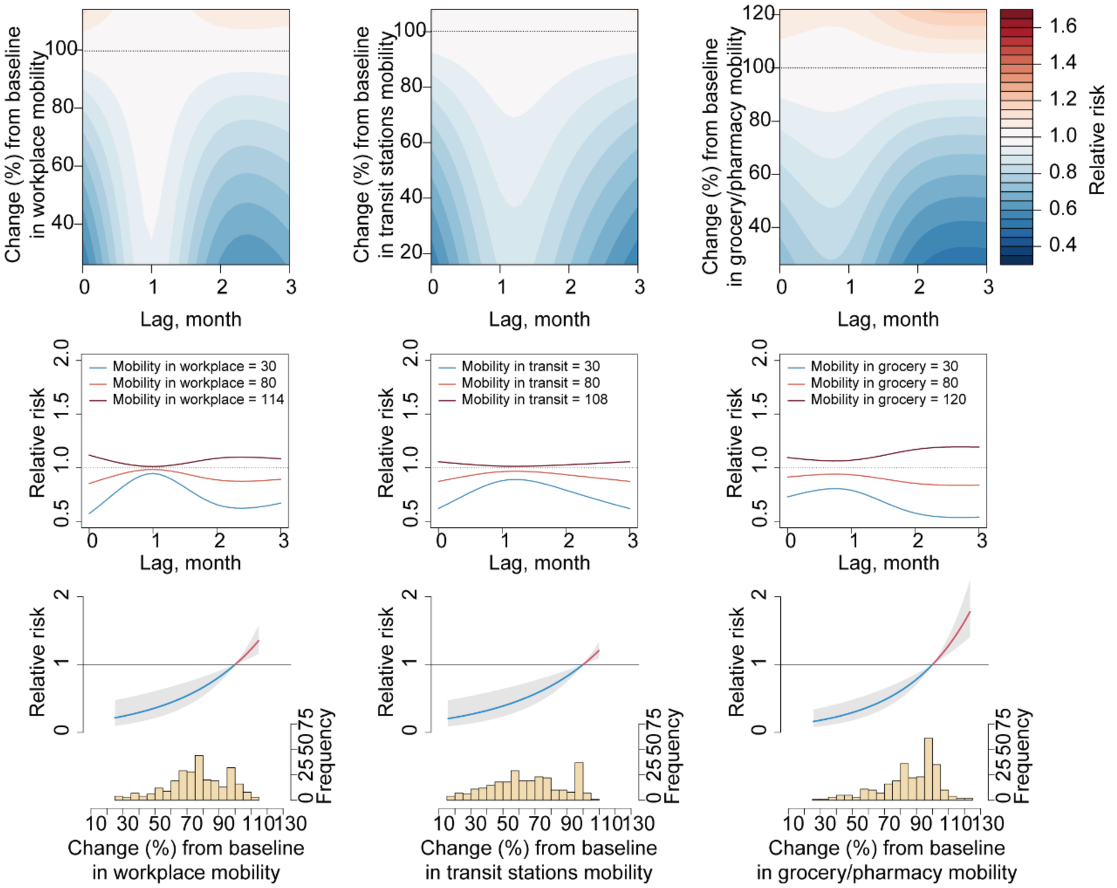

***Figure S14:* The uncertainty in hierarchical cluster analysis of PHSM and human mobility time series.** The height of nodes connecting two variables on the dendrogram represents the degree of similarity. For each cluster in hierarchical clustering, quantities called p-values are calculated via multiscale bootstrap resampling. P-value of a cluster is a value between 0 and 1, which indicates how strong the cluster is supported by data. Red values are AU (Approximately Unbiased) p-values, and green values are BP (Bootstrap Probability) values. AU p-value, which is computed by multiscale bootstrap resampling, is a better approximation to unbiased p-value than BP value computed by normal bootstrap resampling. Clusters with AU larger than 95% are highlighted by rectangles, which are strongly supported by data.

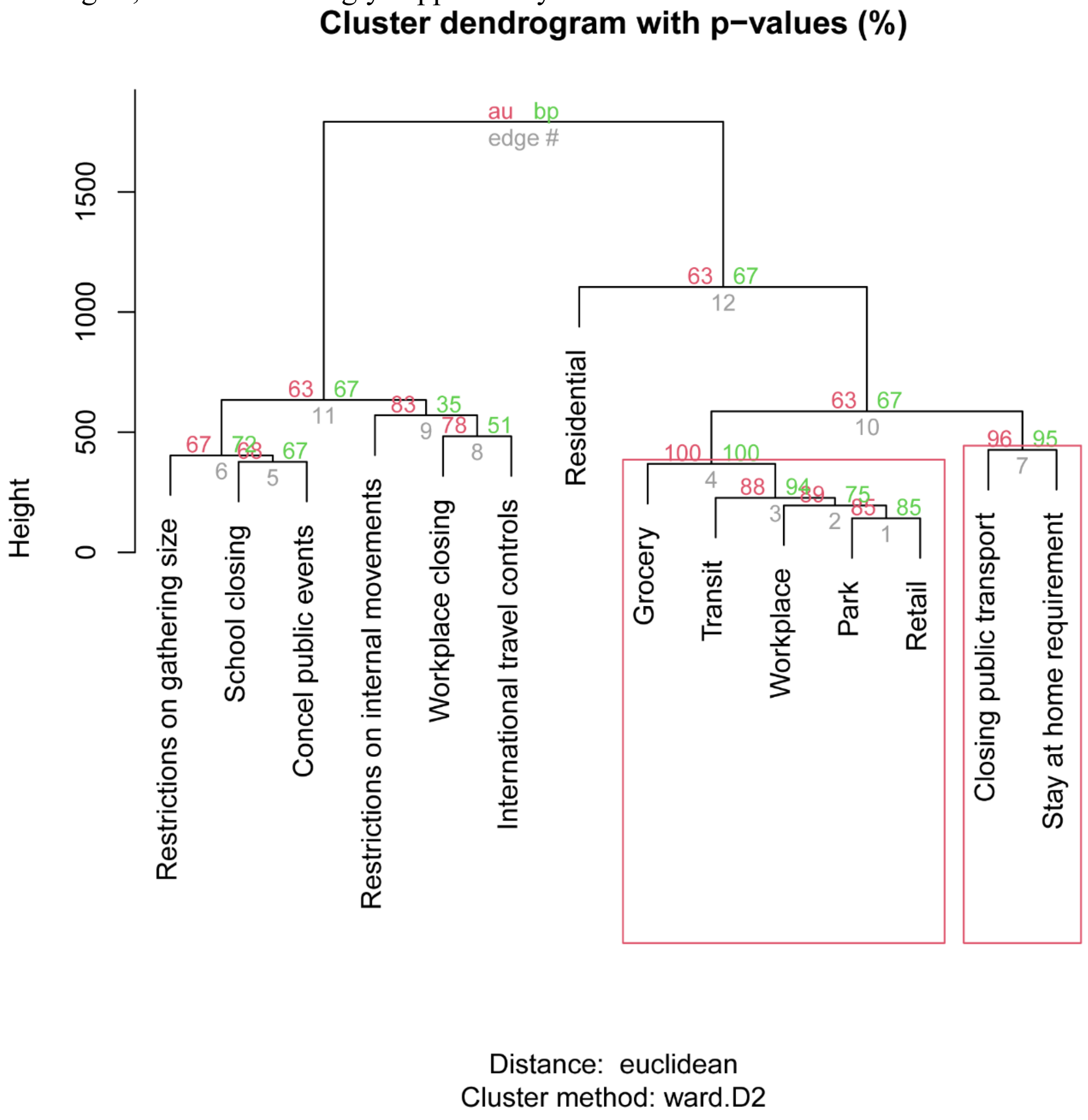

Figure S15: **Heat map of correlations between variables in the Latin America and Southeast Asia, 2020.** C1 - C8 is 8 specific indicators of the containment and closure policies, namely “school closing”, “workplace closing”, “cancelling of public events”, “restrictions on gathering sizes”, “closure of public transport”, “stay at home requirements”, “restrictions on internal movement” and “international travel controls”. Red indicates a positive correlation between the two variables, blue indicates a negative correlation between the two variables, and the numbers in each grid indicate correlation coefficients.

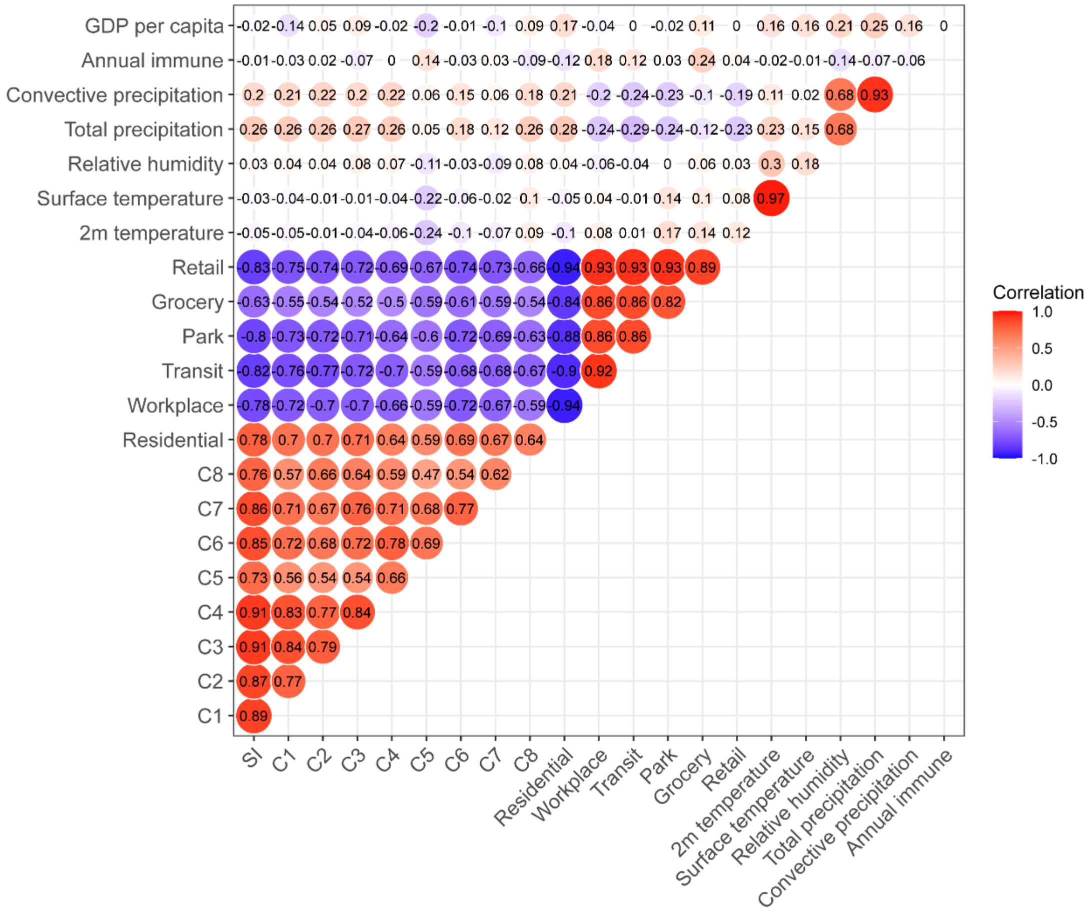

Figure S16: Distributions of residuals between model prediction and observed dengue incidence rate (DIR). The residuals between "historical" and "intervention" model prediction and observed DIR per 100,000 population from January to December for the 23 countries in Latin America and Southeast Asia.

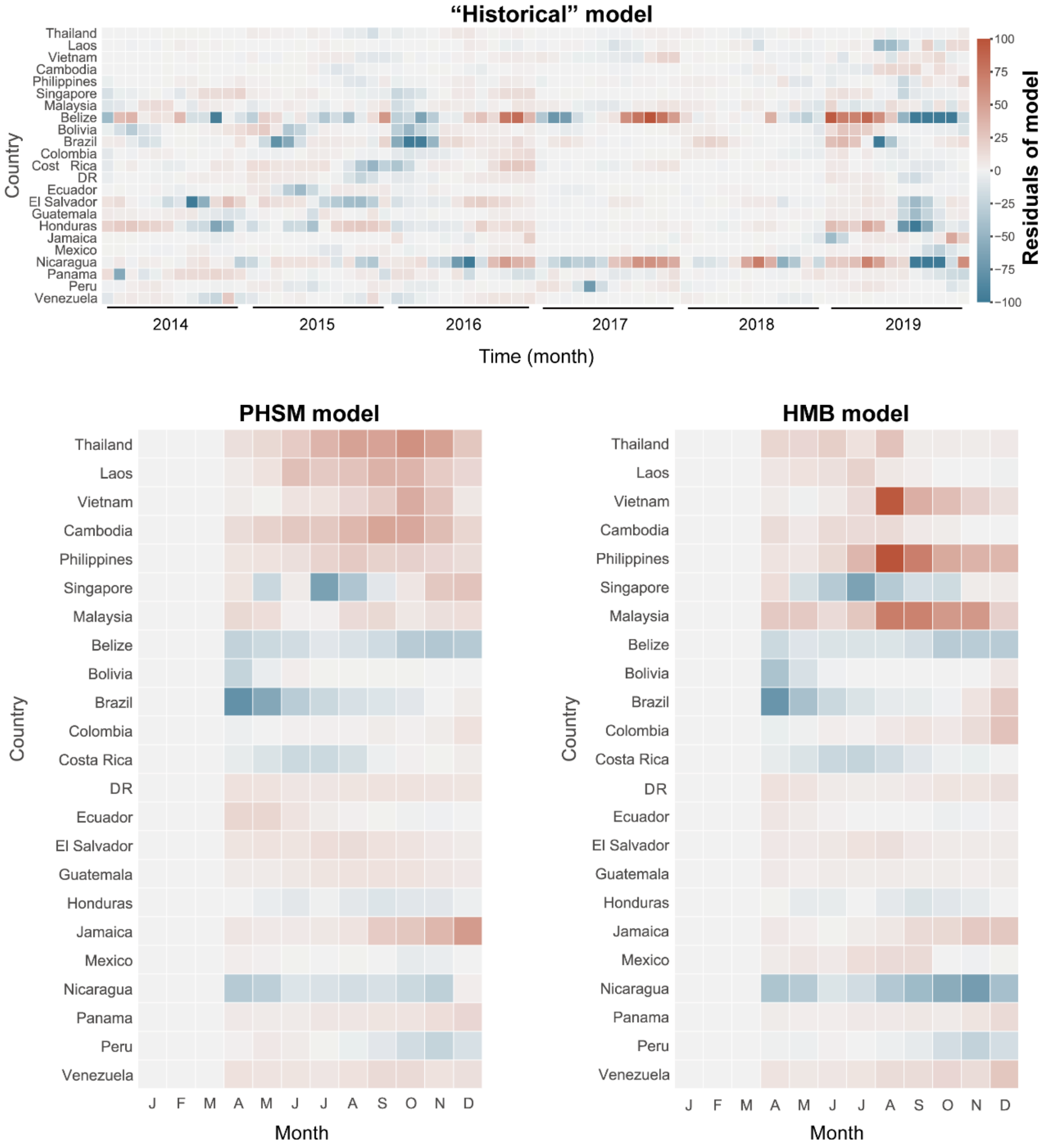

Figure S17: **Proportion of dengue cases averted by PHSM/ human mobility in each country and region at different ranges.**

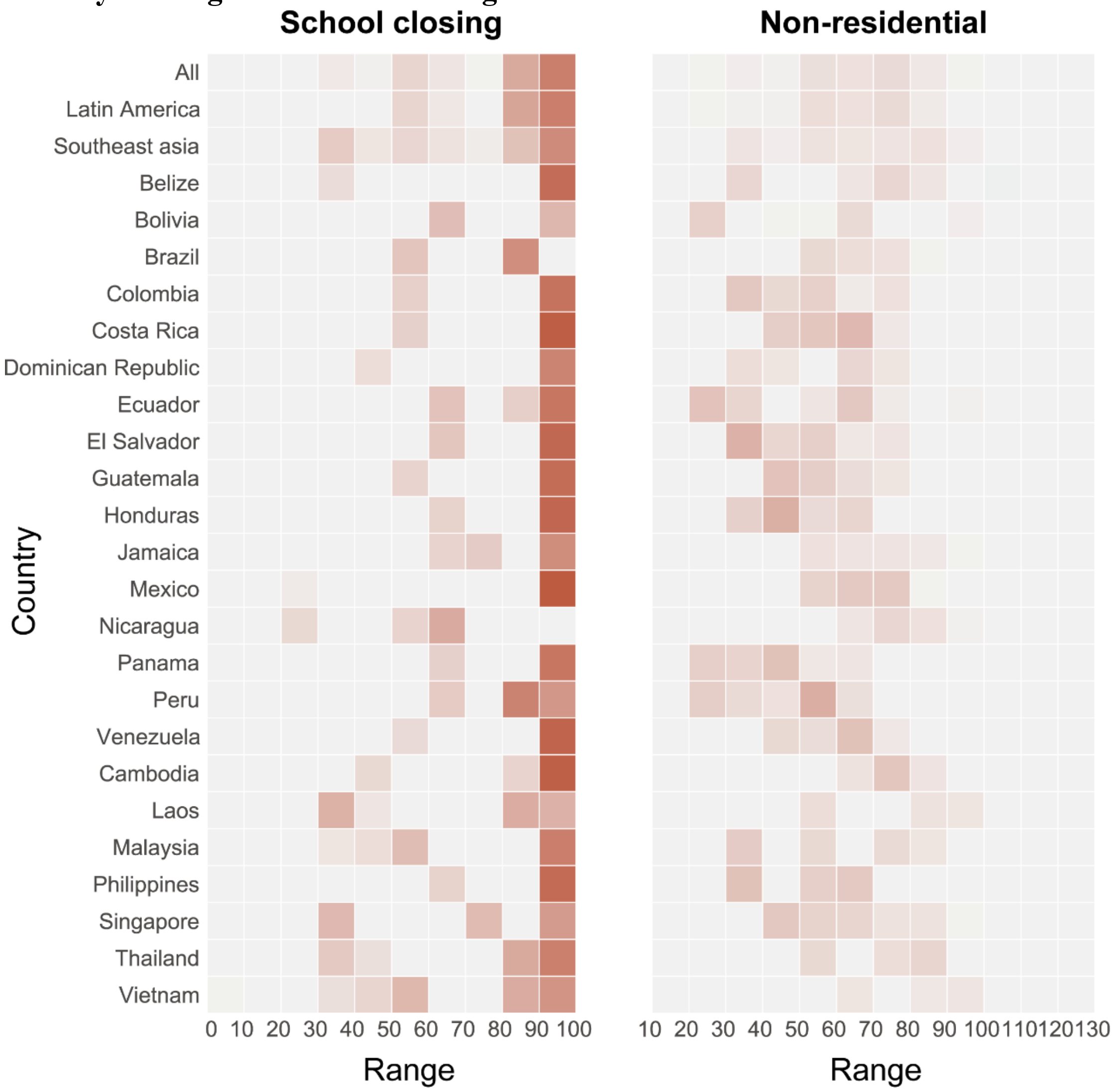

Figure S18: **Predicted and observed monthly dengue cases from the "historical" model.**

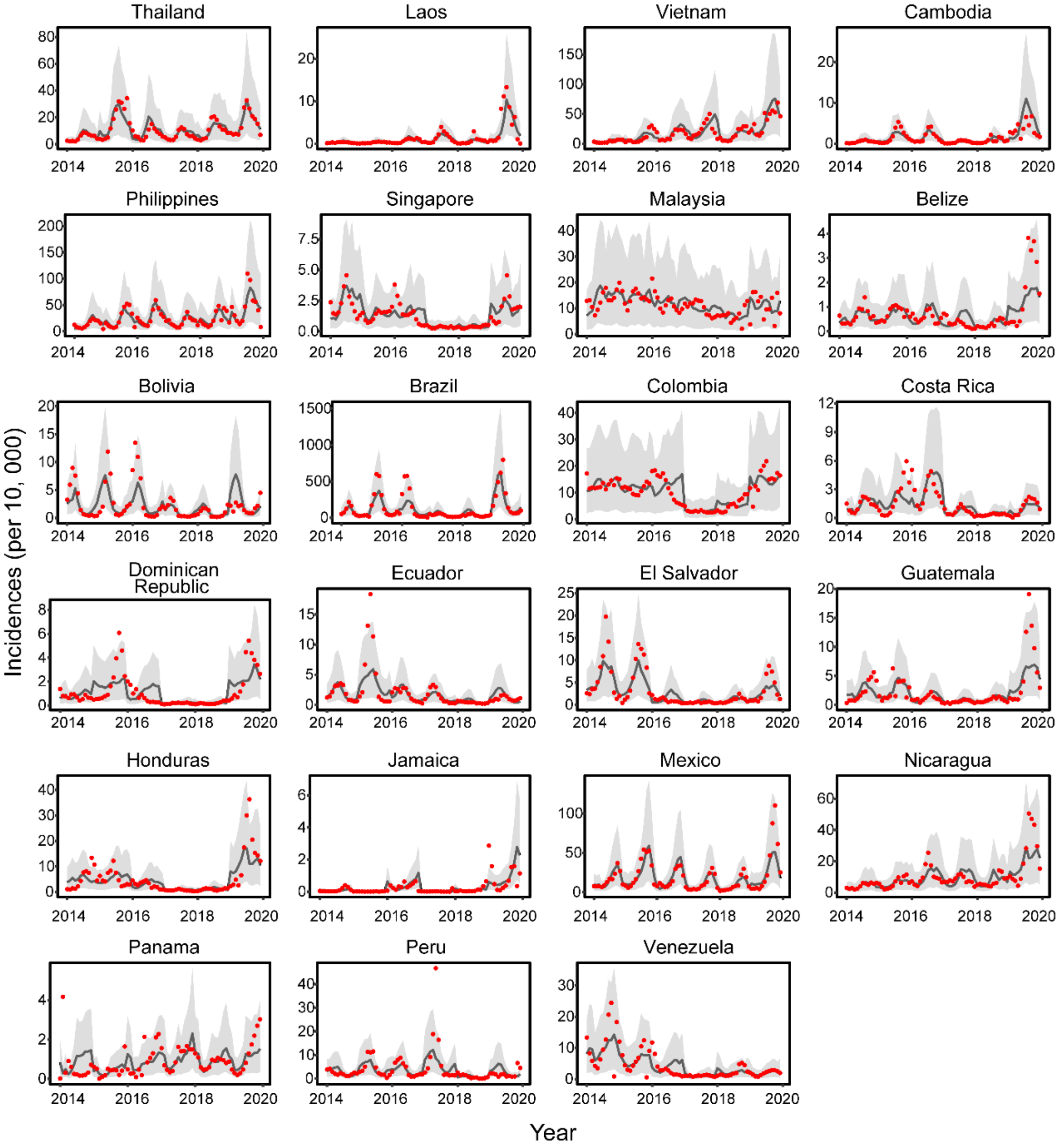

Figure S19: **Predicted and estimated case numbers for the example year 2018 when the historical model was fit to monthly dengue case data 2015-2017.**

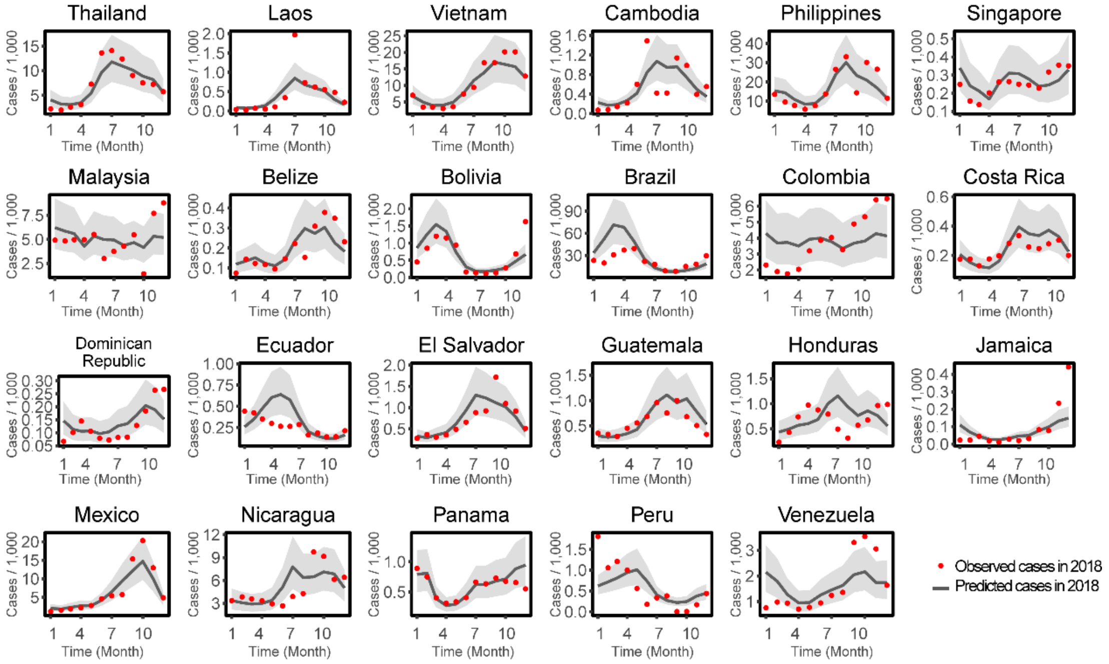

Figure S20: **Predicted and estimated case numbers for the example year 2020 when the historical model was fit to monthly dengue case data 2014-2019.**

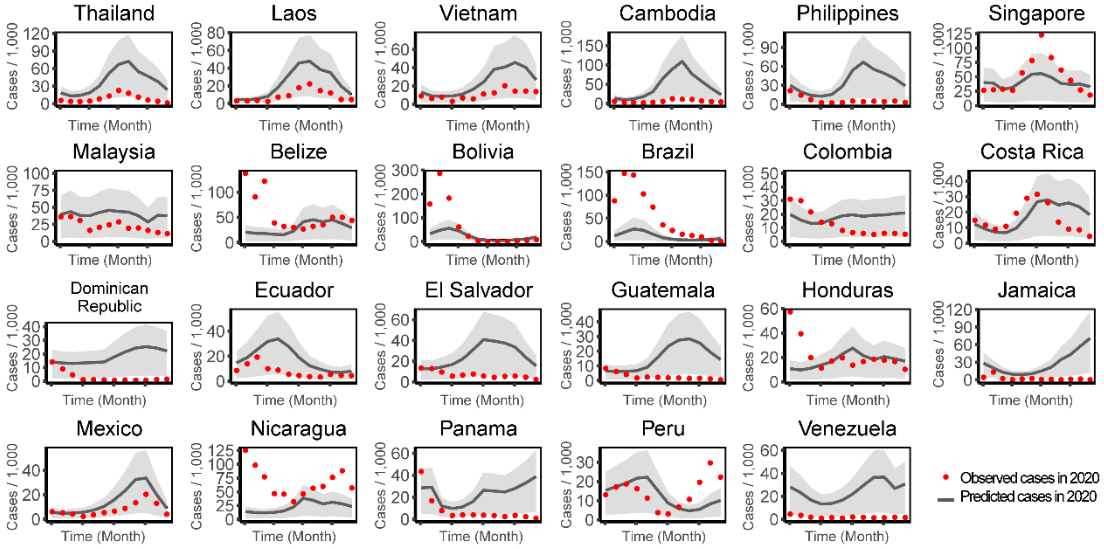

Members of the CMMID working group:

Simon R Procter, Kerry LM Wong, Joel Hellewell, Nicholas G. Davies, Christopher I Jarvis, Ciara V McCarthy, Graham Medley, Sophie R Meakin, Alicia Rosello, Emilie Finch, Rachel Lowe, Carl A B Pearson, Samuel Clifford, Billy J Quilty, Stefan Flasche, Hamish P Gibbs, Lloyd A C Chapman, Katherine E. Atkins, David Hodgson, Rosanna C Barnard, Timothy W Russell, Petra Klepac, Yalda Jafari, Rosalind M Eggo, Paul Mee, Matthew Quaife, Akira Endo, Sebastian Funk, Stéphane Hué, Adam J Kucharski, W John Edmunds, Kathleen O'Reilly, Rachael Pung, C Julian Villabona-Arenas, Amy Gimma, Kaja Abbas, Kiesha Prem, Gwenan M Knight, Fiona Yueqian Sun, William Waites, James D Munday, Mihaly Koltai, Frank G Sandmann, Damien C Tully

Additional funding acknowledgements for CMMID working group members:

Author	Funding
SRP	(B&MGF: INV-016832)
KLM	(European Commission: 101003688)
JH	(Wellcome Trust: 210758/Z/18/Z)
NGD	(NIHR: NIHR200929, UK MRC: MC_PC_19065)
CIJ	(Global Challenges Research Fund: ES/P010873/1)
CVM	(NIHR: NIHR200929)
GFM	(B&MGF: NTD Modelling Consortium OPP1184344)
SRM	(Wellcome Trust: 210758/Z/18/Z)
AR	(NIHR: PR-OD-1017-20002)
EF	(MRC: MR/N013638/1)
RL	(Royal Society: Dorothy Hodgkin Fellowship)
CABP	(B&MGF: NTD Modelling Consortium OPP1184344, FCDO/Wellcome Trust: Epidemic Preparedness Coronavirus research programme 221303/Z/20/Z)
SC	(Wellcome Trust: 208812/Z/17/Z, UK MRC: MC_PC_19065)
BJQ	(NIHR: 16/137/109, NIHR: 16/136/46, B&MGF: OPP1139859)
SFla	(Wellcome Trust: 208812/Z/17/Z)
HPG	(EDCTP2: RIA2020EF-2983-CSIGN, UK DHSC/UK Aid/NIHR: PR-OD-1017-20001)
LACC	(NIHR: NIHR200908)
KEA	(ERC: SG 757688)
DH	(NIHR: 1R01AI141534-01A1)
RCB	(European Commission: 101003688)
TWR	(Wellcome Trust: 206250/Z/17/Z)
PK	(Royal Society: RP\EA\180004, European Commission: 101003688)
YJ	(UKRI: MR/V028456/1)
RME	(HDR UK: MR/S003975/1, UK MRC: MC_PC_19065, NIHR: NIHR200908)
PM	CADDE (MR/S0195/1 & FAPESP 18/14389-0)
MQ	(ERC Starting Grant: #757699, B&MGF: INV-001754)
AE	(Nakajima Foundation)
SFunk	(Wellcome Trust: 210758/Z/18/Z)
AJK	(Wellcome Trust: 206250/Z/17/Z, NIHR: NIHR200908)

WJE	(European Commission: 101003688, UK MRC: MC_PC_19065, NIHR: PR-OD-1017-20002)
KO'R	(B&MGF: OPP1191821)
RP	(Singapore Ministry of Health)
CJVA	(ERC: SG 757688)
AG	(European Commission: 101003688)
KA	(BMGF: INV-016832; OPP1157270)
KP	(B&MGF: INV-003174, European Commission: 101003688)
GMK	(UK MRC: MR/P014658/1)
FYS	(NIHR: 16/137/109)
WW	(MRC: MR/V027956/1)
JDM	(Wellcome Trust: 210758/Z/18/Z)
MK	(Wellcome Trust: 221303/Z/20/Z)
FGS	(NIHR: NIHR200929)

References

1. Hale T, Angrist N, Goldszmidt R, et al. A global panel database of pandemic policies (Oxford COVID-19 Government Response Tracker). *Nat Hum Behav* 2021; **5**(4): 529-38.
2. Lowe R, Barcellos C, Coelho CA, et al. Dengue outlook for the World Cup in Brazil: an early warning model framework driven by real-time seasonal climate forecasts. *Lancet Infect Dis* 2014; **14**(7): 619-26.
3. Schrödle B, Held L. A primer on disease mapping and ecological regression using INLA. *Computation Stat* 2011; **26**(2): 241-58.
4. Riebler A, Sørbye SH, Simpson D, Rue H. An intuitive Bayesian spatial model for disease mapping that accounts for scaling. *Stat Methods Med Res* 2016; **25**(4): 1145-65.
5. Gasparrini A. Distributed Lag Linear and Non-Linear Models in R: The Package dlnm. *J Stat Softw* 2011; **43**(8): 1-20.
6. Gasparrini A, Armstrong B, Kenward MG. Distributed lag non-linear models. *Stat Med* 2010; **29**(21): 2224-34.
7. Naish S, Dale P, Mackenzie JS, McBride J, Mengersen K, Tong S. Climate change and dengue: a critical and systematic review of quantitative modelling approaches. *BMC Infect Dis* 2014; **14**: 167.
8. Lowe R, Bailey TC, Stephenson DB, et al. The development of an early warning system for climate-sensitive disease risk with a focus on dengue epidemics in Southeast Brazil. *Stat Med* 2013; **32**(5): 864-83.
9. Hii YL, Rocklöv J, Wall S, Ng LC, Tang CS, Ng N. Optimal lead time for dengue forecast. *PLoS Negl Trop Dis* 2012; **6**(10): e1848.
